# Siah2 control of T-regulatory cells limits anti-tumor immunity

**DOI:** 10.1038/s41467-019-13826-7

**Published:** 2020-01-07

**Authors:** Marzia Scortegagna, Kathryn Hockemeyer, Igor Dolgalev, Joanna Poźniak, Florian Rambow, Yan Li, Yongmei Feng, Roberto Tinoco, Dennis C. Otero, Tongwu Zhang, Kevin Brown, Marcus Bosenberg, Linda M. Bradley, Jean-Christophe Marine, Ioannis Aifantis, Ze’ev A. Ronai

**Affiliations:** 1 Cancer Center, Sanford Burnham Prebys Medical Discovery Institute, La Jolla, CA 92037 USA; 2 Department of Pathology and Laura and Isaac Perlmutter Cancer Center, NYU School of Medicine, New York, NY 10016 USA; 3 VIB Center for Cancer Biology Laboratory for Molecular Cancer Biology, KU Leuven Oncology Department, Leuven, Belgium; 4 Division of Cancer Epidemiology and Genetics, National Cancer Institute, Bethesda, MD 20892 USA; 5 Departments of Dermatology, Pathology, Yale University, School of Medicine, New Haven, CT 06520 USA; 6 Department of Molecular Biology and Biochemistry, University of California, Irvine, Irvine, CA 92697 USA

**Keywords:** Cancer, Skin cancer, Tumour immunology, Cell biology, Gene regulation in immune cells

## Abstract

Understanding the mechanisms underlying anti-tumor immunity is pivotal for improving immune-based cancer therapies. Here, we report that growth of BRAF-mutant melanoma cells is inhibited, up to complete rejection, in *Siah2*^*−/−*^ mice. Growth-inhibited tumors exhibit increased numbers of intra-tumoral activated T cells and decreased expression of *Ccl17,* *Ccl22*, and *Foxp3*. Marked reduction in Treg proliferation and tumor infiltration coincide with G1 arrest in tumor infiltrated *Siah2*^*−/−*^ Tregs in vivo or following T cell stimulation in culture, attributed to elevated expression of the cyclin-dependent kinase inhibitor p27, a Siah2 substrate. Growth of anti-PD-1 therapy resistant melanoma is effectively inhibited in *Siah2*^*−/−*^ mice subjected to PD-1 blockade, indicating synergy between PD-1 blockade and *Siah2* loss. Low *SIAH2* and *FOXP3* expression is identified in immune responsive human melanoma tumors. Overall, Siah2 regulation of Treg recruitment and cell cycle progression effectively controls melanoma development and Siah2 loss in the host sensitizes melanoma to anti-PD-1 therapy.

## Abstract (web summary)

The ubiquitin ligase Siah2 has been implicated in immune responses. Here, the authors show that Siah2 null immune cells have an increased inflammatory response to inoculated melanoma cells, along with a reduced number of infiltrating immunosuppressive regulatory T cells, resulting in inhibition of tumour growth.

## Introduction

The development of immune checkpoint therapy (ICT), which unleashes an immune response against cancer, is among the most rapid and significant advances in cancer therapy over the past decade^1,2^. While responses to monotherapy are often limited, combining ICT with targeted therapies and, more recently, neoadjuvant therapy has been shown to be more effective and durable, and has been extended to numerous cancer subtypes, impacting the lives of more patients^3–5^. Efforts to identify markers that help stratify responders and those at risk to develop resistance to ICT^6^, will benefit from better understanding of mechanisms underlying immune system regulation and function.

The ubiquitin proteasome system is part of a regulatory cascade that underlies spatial and temporal control of key cellular functions in cell- and tissue-dependent manners. Ubiquitin proteasome signaling controls key immune regulatory functions, including pattern recognition receptor signaling, toll-like receptor signaling^7,8^, RIG1-like receptor signaling^9,10^, Nod-like receptor signaling^11^, STING signaling^12^, dendritic cell (DC) maturation and function^13–15^, and T cell activation, tolerance, and autoimmunity^16^. Understanding mechanisms underlying the role of ubiquitin ligases in the control of tumor immunity is expected to identify markers for patient stratification, and targets directing therapeutic modalities.

The E3 ubiquitin ligase Siah2 functions to control a number of fundamental cellular processes, including hypoxia^17,18^, the unfolded protein response (UPR^19^), cell junction integrity^20^, mitochondrial dynamics^21^, intracellular signaling^22^, cellular metabolism^23^, and cell proliferation^24–27^. While these activities are associated with immune cell function, direct evidence for Siah2 regulation of anti-tumor immunity has been lacking. Global functional profiling of the human ubiquitinome identified Siah2 as controlling type I interferon signaling^28^, pointing to a possible role for Siah2 in the immune response. Here, using a genetic mouse model of global Siah2 deletion, we show that Siah2 functions in intratumoral recruitment and cell cycle control of T cells with the most notable impact on T regulatory cells (Tregs), supporting a function in anti-tumor immunity.

Key in understanding the effectiveness of anti-tumor immunity is the balance between tumor-infiltrating active and suppressive T cells. While cytokines and chemokines play key role in the recruitment, proliferation, and function of distinct T cell populations within tumors, other factors, intrinsic to individual subpopulations define their ability to withstand the harsh intratumoral environment, often characterized by low oxygen tension and limited availability of nutrients. Notably, the suppressive T cell population, Treg cells, has an advantage in sustaining the intratumoral milieu, reflected in greater propensity to proliferate and exert suppressive function under harsh environmental conditions, compared with the effector T cells^29,30^. Thus, selective suppression of Tregs is expected to enhance anti-tumor immunity. Here, we demonstrate that via its regulation of p27 stability in stimulated T cells, Siah2 controls T cell proliferation, which affects the availability of intratumoral Treg, but not effector T cells, thereby enhancing effectiveness of the cytotoxic T cell and overall anti-tumor immunity.

## Results

### Enhanced anti-tumor immunity in melanoma grown in *Siah2*^*−*/*−*^ mice

**Fig. 1:**
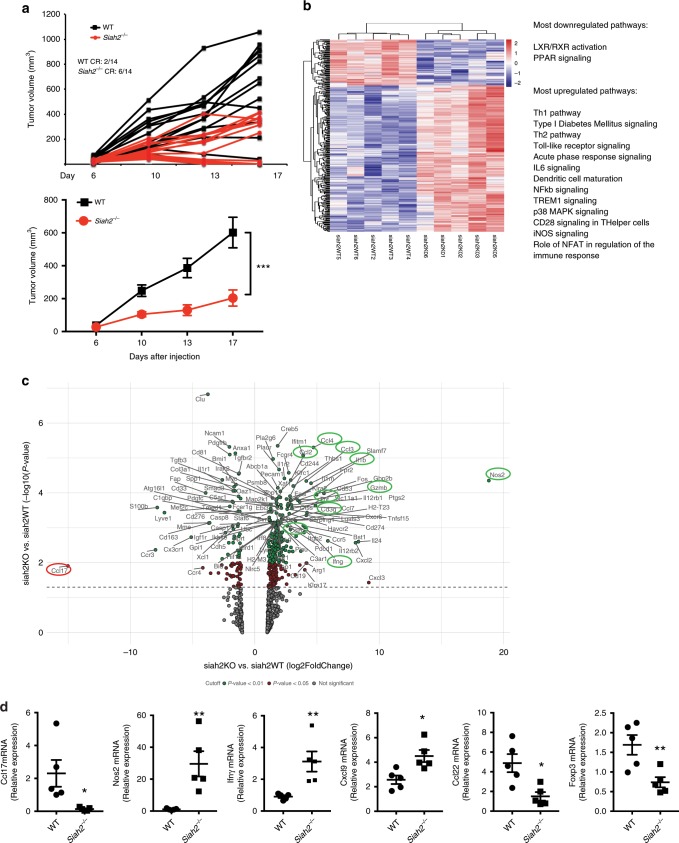
Siah2-deficient mice limits melanoma growth. **a** YUMMER1.7 melanoma cells (400,000) were injected s.c. into the flank of 5–7-weeks-old *Siah2* WT or *Siah2*^*−/−*^ male mice, and mean (lower panel) and individual (upper panel) tumor growth (volume) was measured over time (*N* = 14 for both genotypes). Analysis shows complete regression (CR) at study termination. **b** NanoString analysis of PanCancer Immune Profiling genes in tumors from WT and *Siah2*^*−/−*^ mice (*N* = 5 for both genotypes). Heat map shows the most upregulated and downregulated pathways in *Siah2*^*−/−*^ mice based on comparisons of YUMMER1.7 tumors (*n* = 5). A total of 364 differentially expressed genes were found, with 87 downregulated and 277 upregulated in *Siah2*^*−/−*^ tumors. Analysis was performed 10 days after tumor injection. Cutoff applied: *P* < 0.05. **c** Volcano plot from NanoString analysis showing genes differentially expressed in *Siah2*^*−/−*^ versus WT tumors. Cutoff is color coded: green = *P* < 0.01, red = *P* < 0.05, grey = not significant. **d** q-RTPCR analysis of indicated mRNAs from tumors collected 10 days after injection. *N* = 5, both genotypes. Data in **a** and **d** are presented as means ± s.e.m. Data in **a** were analyzed by two-way ANOVA. Data in **d** were analyzed by unpaired *t*-test. **P < 0.005 and *P < 0.05 compared with WT.

To evaluate Siah2 function in the tumor environment, we injected cells of the BRAF-mutant melanoma line YUMMER1.7 into syngeneic wild-type (WT) or *Siah2*^*−/−*^ mice. The YUMMER1.7 line carries a high somatic mutation burden and is more immunogenic than the parental YUMM1.7 line^31,32^. Growth of YUMMER1.7 cells was largely attenuated in *Siah2*^*−/−*^ relative to WT mice, (Fig. 1a), with no obvious changes in gross tumor morphology or melanoma marker expression (Supplementary Fig. 1a, b). Notably, 6 of 14 tumors (42%) grown in *Siah2*^*−/−*^ mice exhibited complete regression as compared with 2/14 (14%) tumors in WT mice (Fig. 1a). While melanoma development in the first few days following tumor cell inoculation was similar in both the WT and *Siah2*^*−/−*^ mice, within 10–14 days tumors began to regress in the *Siah2*^*−/−*^ mice, while they continued growing in the WT genotype. Increasing the number of tumor cells inoculated (from 4 × 10^5^ to 1 × 10^6^) abrogated the tumor rejection phenotype in *Siah2*^*−/−*^ mice (Supplementary Fig. 1c), suggesting that tumor burden is a critical determinant of effective Siah2-dependent immune cell function.

To map possible changes in the tumor microenvironment of *Siah2*^*−/−*^ mice that may contribute to tumor growth inhibition, we performed RNA sequencing (RNAseq) on both WT or *Siah2*^*−/−*^ tumors. An enhanced inflammatory gene signature was identified in tumors harvested from *Siah2*^*−/−*^ relative to WT mice, a signature characterized by upregulation of genes implicated in the Th1 pathway and NOS2 signaling (Supplementary Fig. 1d). To further map the effect of Siah2 on immune signaling, we performed PanCancer Immune Profiling using the NanoString technology. Common to both RNAseq and NanoString analyses were increased expression of genes that function in immune cell inflammatory and effector phenotypes (among them, *Gzmb*, *Gzma*, *Ifn*γ, and *Tnfα*; Fig. 1b–d, Supplementary Fig. 1d) and increased expression of genes associated with T cell identity (i.e., the NFAT activation pathway genes *Cd3*γ, *Cd3δ*, and *Cd8α*; Fig. 1b, c, Supplementary Fig. 1d). Both analyses also showed increased expression of chemokines (*Ccl4, Ccl3, Ccl2, and Ccl5*) and cytokines (*Il1b* and *Cxcl9*; Fig. 1c, d, Supplementary Fig. 1d) in tumors from *Siah2*^*−/−*^ mice. *Nos2* was among the most upregulated genes in *Siah2*^*−/−*^ mice, while *Ccl17*, which contribute to recruitment of CCR4^+^ Tregs to tumors and thus to immunosuppression^33,34^, was among the most downregulated (Fig. 1c, d). Diminished attraction of Tregs to tumors resulting from reduced *Ccl17/22* levels is consistent with improved anti-tumor immunity and attenuated tumor growth. Accordingly, both RNAseq and NanoString analyses revealed significantly reduced expression of *Foxp3*, a Treg marker, in tumors harvested from *Siah2*^*−/−*^ mice, a decrease confirmed by quantitative PCR (qPCR) analysis (Fig. 1d). Overall, these findings reveal an increased inflammatory and activated immune phenotype in the *Siah2*^*−/−*^ tumor immune environment, concomitant with reduced Treg infiltration.

### Increased T effector cells and fewer Tregs in *Siah2*^*−*/*−*^ mice grown tumors

**Fig. 2:**
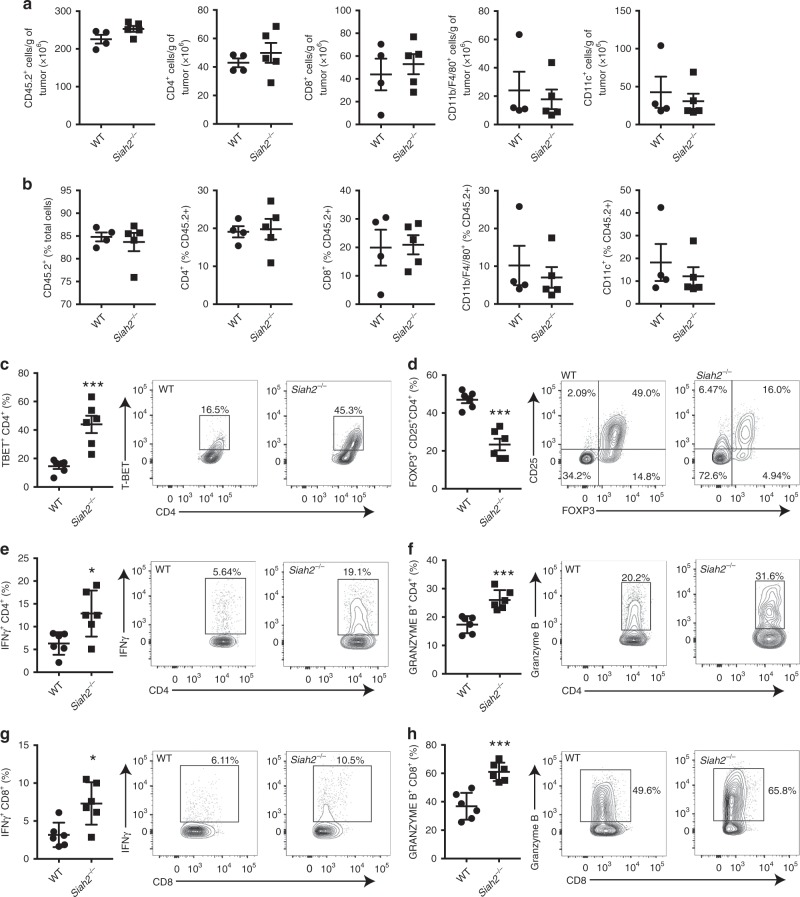
Altered intratumoral effector T cells and Tregs in * Siah2*^*−*/*−*^ mice. **a**, **b** Quantification of tumor-infiltrating immune cells (CD45.2^+^), CD4^+^ and CD8^+^ T cells, CD11b^+^/F4/80^+^, and CD11c^+^ cells on day 11 after YUMMER1.7 cell injection. Quantification is reported as total number/g tumor **a**, and as percentage **b** of indicated immune cells among total cells (for the graph in **b** analyzing frequency of CD45.2^+^), or CD45.2^+^ cells (for other graphs in **b**; *n* = 4 for WT; *n* = 5 for KO). **c**, **d** Percentage of tumor-infiltrating T-bet^+^ **c** and FOXP3^+^ **d** cells within the CD4^+^ T cell population at day 11 after tumor inoculation (*n* = 5 for WT; *n* = 6 for *Siah2*^*−/−*^). **e**–**h** Frequencies of tumor-infiltrating IFN-γ- and granzyme B-expressing CD4^+^ **e**, **f** or CD8^+^ **g**, **h** T cells 11 days after tumor cell inoculation and following stimulation overnight in vitro with melanoma peptides (*n* = 6) **e**–**h**; mean ± s.e.m. Data were analyzed by unpaired *t*-test. ***P < 0.0005, **P < 0.005, and *P < 0.05 compared with WT.

We next compared the type and quantity of infiltrating immune cells in tumors grown in *Siah2*^*−/−*^ and WT littermates. Flow cytometry analysis performed on tumors collected 11 days after melanoma cell inoculation, a time point when tumors begin to shrink in *Siah2*^*−/−*^ mice (Supplementary Fig. 2a) revealed a comparable number (Fig. 2a) or proportion (Fig. 2b) of CD45.2^+^, CD4^+^, CD8^+^, CD11b^+^ F4/80^+^, CD11c^+^, and CD11b^+^GR1^+^ cells in both genotypes (Fig. 2a, b, Supplementary Fig. 2b). However, a 3-fold increase in the T-bet^+^ cell population and a 2-fold decrease in FOXP3^+^CD25^+^ cells within the CD4^+^ population was seen in tumors grown in *Siah2*^*−/−*^ mice as compared to WT mice (Fig. 2c, d), while WT and *Siah2*^*−/−*^ tumors showed comparable expression of FOXP3 within the Treg cell population (Supplementary Fig. 2c). These findings suggest that reduced infiltration of Treg cells is accompanied by increased infiltration of T effector cells. These observations led us to assess possible changes in the Treg population, relative to other tumor-infiltrating immune cell types in *Siah2*^*−/−*^ mice. Immunohistochemistry confirmed a significant decrease in the number of FOXP3^+^ cells, but not in the number of CD3^+^ cells, within tumors grown in *Siah2*^*−/−*^ mice (Supplementary Fig. 2d, e). Furthermore, FOXP3-negative T cells exhibited an activated effector phenotype, as reflected by an increase in IFNγ and granzyme B in both CD4^+^ and CD8^+^ T cell populations that had infiltrated tumors in *Siah2*^*−/−*^ and WT mice (Fig. 2e–h). A relative increase in the number of T-bet^+^ Th1 CD4^+^ cells was seen in *Siah2*^*−/−*^ mice (Fig. 2c), which can be cytotoxic and produce IFNγ^35^. *Siah2*^*−/−*^ mice also showed increased NOS2 expression in the macrophage population (Supplementary Fig. 2f), further suggesting that *Siah2*^*−/−*^ loss promotes an inflammatory phenotype, confirming RNAseq and NanoString data.

We next assessed whether the less immunogenic YUMM1.7 cells also exhibit the unique tumor infiltration signature. YUMM1.7 tumors that were collected 11 days after inoculation were smaller in *Siah2*^*−/−*^ relative to WT mice (Supplementary Fig. 2g), and exhibited increased infiltration of CD4^+^ cells expressing IFNγ (Supplementary Fig. 2h). YUMM1.7 tumors grown in *Siah2*^*−/−*^ mice also showed decreased frequency of tumor-infiltrating Tregs (Supplementary Fig. 2i) as well as macrophages positive for CD206 (Supplementary Fig. 2j), a pattern recognition receptor associated with tumor immunosuppression. These phenotypes were accompanied with increased frequency of the DC and macrophages expressing costimulatory receptor CD80^+^ DC (Supplementary Fig. 2k). Like YUMMER1.7 tumors, percentages of tumor-infiltrating immune cells were comparable in YUMM1.7 tumors from *Siah2*^*−/−*^ and WT mice (Supplementary Fig. 2l). Collectively, these findings suggest that with the exception of Tregs, Siah2 regulates activity but not the frequency of intratumoral immune cells.

### Tumor-infiltrating cells inhibit tumor growth in Siah2^*−*/*−*^ mice

**Fig. 3:**
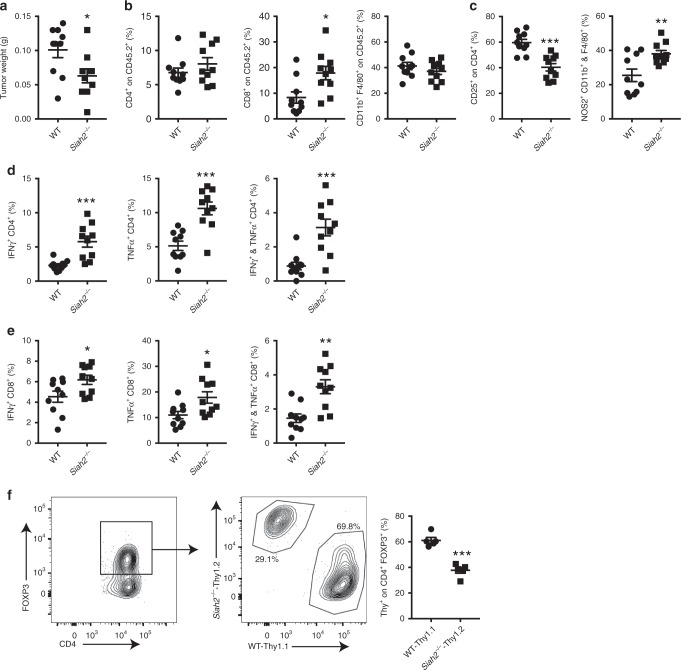
Decreased tumor growth in *Siah2*^*−*/*−*^ BM-transplanted mice. **a** Weight of tumors collected 14 days after YUMMER1.7 cell inoculation into lethally irradiated mice transplanted with bone marrow (BM) from WT or *Siah2*^*−/−*^ mice (*n* = 10). **b** Frequencies of tumor-infiltrating CD4^+^, CD8^+^ T cells, and CD11b^+^/F4/80^+^ cells among CD45^+^ cells on day 14 after tumor cell injection (*n* = 10). **c** Frequencies of CD25^+^ cells among CD4^+^ cells and NOS2-producing CD11b/F4/80-positive cells (*n* = 10). **d**, **e** Frequencies of tumor-infiltrating TNF-α- and IFN-γ-producing CD4^+^ **d** and CD8^+^ **e** T cells on day 14 after tumor inoculation and following overnight stimulation in vitro with melanoma peptides (*n* = 10). **f** A 1:1 mixture of BM from Thy1.1^+^ CD45.2^+^ WT and Thy1.2^+^ CD45.2^+^ *Siah2*^*−/−*^ mice was injected into lethally irradiated CD45.1^+^ mice. YUMMER1.7 cells were injected 8 weeks after reconstitution and tumors collected 14 days later. CD4^+^ Foxp3^+^ cells were gated and percentages of WT Thy1.1-positive and *Siah2*^*−/−*^ Thy1.2^+^ cells were determined (*n* = 5); mean ± s.e.m. Data were analyzed by unpaired *t*-test. ***P < 0.0005, **P < 0.005, and *P < 0.05 compared with WT.

To determine whether Siah2 regulates cell autonomous tumor infiltrated immune cells, bone marrow (BM) cells from WT or *Siah2*^*−/−*^ mice were transplanted into irradiated WT recipient mice, and 8 weeks later both groups were injected subcutaneously (s.c.) with YUMMER1.7 melanoma cells. Smaller tumors were identified in mice transplanted with *Siah2*^*−/−*^ relative to WT BM (Fig. 3a). While no differences were observed in intratumoral infiltration of CD4^+^ or CD11b^+^ F4/80^+^ cells, a notable increase in the number of CD8^+^ cells and a marked decrease in Tregs were seen in *Siah2*^*−/−*^ BM-transplanted mice inoculated with YUMMER1.7 cells, which was accompanied by increased NOS2 expression within tumor-infiltrating macrophages (Fig. 3b, c).

Similar to our observations in *Siah2*^*−/−*^ mice, intratumoral T cells in mice transplanted with BM from *Siah2*^*−/−*^ mice showed increased effector function, as reflected by increased levels of IFNγ and TNFα produced by both CD4^+^ (Fig. 3d) and CD8^+^ (Fig. 3e) T cell populations relative to mice transplanted with WT BM. These data confirm that intratumoral immune cells in *Siah2*^*−/−*^ mice are intrinsically more activated and exhibit more pronounced inflammatory phenotypes.

To further assess how Siah2 regulates the intratumoral Treg population, transplant experiments were performed, in which lethally irradiated CD45.1 mice were injected with a 1:1 mixture of BM cells from WT Thy1.1^+^CD45.2^+^ and *Siah2*^*−/−*^ Thy1.2^+^CD45.2^+^ mice. Eight weeks later, mice were inoculated with YUMMER1.7 cells and tumors were collected 14 days later. Fluorescence-activated cell sorting (FACS) analysis of tumor cell populations revealed that among Foxp3^+^ cells, 40% were *Siah2*^*−/−*^ and 60% were *Siah2* WT (Fig. 3f). Of note, prior to tumor injection blood samples contained a comparable distribution of WT Thy1.1^+^ CD45.2^+^ and *Siah2*^*−/−*^ Thy1.2^+^ CD45.2^+^ in the CD4^+^ and CD8^+^ pools (Supplementary Fig. 3). Overall, these findings indicate that *Siah2* regulates a Tregs intrinsic mechanism that promotes their intratumoral cell numbers, therefore increasing Treg-dependent immunosuppression within tumors.

### Reduced Tregs in Siah2^*−*/*−*^grown tumors by single-cell analysis

**Fig. 4:**
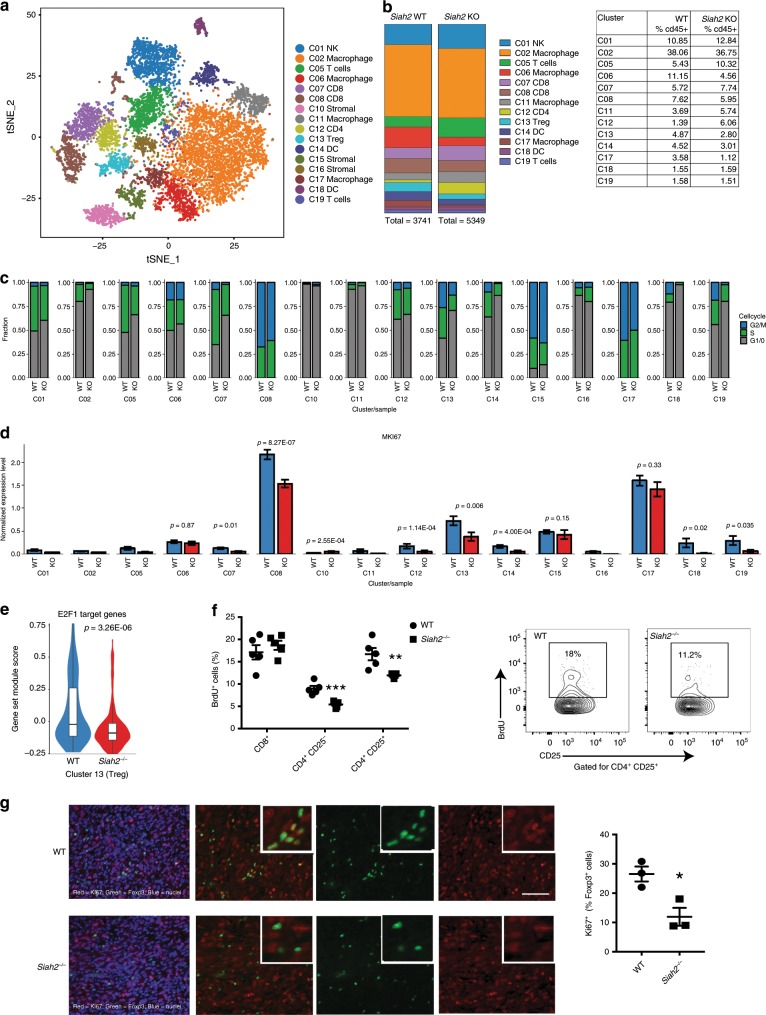
Reduced proliferation of tumor-infiltrating Tregs in *Siah2*^*−*/*−*^ mice. **a** *t*-SNE plot of CD45^+^ cells from melanoma tumors collected 11 days after inoculation of YUMMER1.7 cells into WT or *Siah2*^*−/−*^ mice, showing different clusters. **b** Color-coded bars (left) and table (right) represent proportions of cells in each cluster within CD45^+^ clusters from WT and *Siah2*^*−/−*^ tumors. **c** Bar graphs showing cell cycle status of T cells, based on single-cell RNAseq in *Siah2* WT and *Siah2*^*−/−*^ cells. **d** Expression of Ki67 (MKi67) mRNA identified by single-cell RNAseq within indicated clusters in both genotypes. **e** Violin plot comparing expression levels of E2F1-regulated genes based on single-cell RNAseq. **f** BrdU was injected into *Siah2* WT and *Siah2*^*−/−*^ mice-bearing YUMMER1.7 melanoma tumors 16 h before tumor collection. Shown is BrdU incorporation by T cells, as determined by flow cytometry (*n* = 5). **g** Ki67(red)/Foxp3(green) staining of tumors from *Siah2* WT or *Siah2*^*−/−*^ mice analyzed 11 days after melanoma cell injection (left panels), plus quantification (right; *n* = 3). Scale bar, 100 μm; mean ± s.e.m. Data were analyzed by unpaired *t*-test in **f** and **g**, and by Wilcoxon rank-sum test in **d** and **e**. **P < 0.0005 and *P < 0.05 compared with WT.

To further assess Siah2-dependent changes in tumor-infiltrating immune cell populations, we carried out single-cell RNAseq analysis of CD45^+^ cells from YUMMER1.7 tumors grown in WT or *Siah2*^*−/−*^ mice. Data were analyzed by unsupervised density-based clustering using the *t*-distributed Stochastic Neighbor Embedding (*t*-SNE) algorithm, which enabled partition of cell populations into 16 different clusters (Fig. 4a, Supplementary Fig. 4a). Clusters 7 and 8, both containing CD8^+^ T cells, showed increased expression of granzyme B, while IFNγ expression increased in cluster 8 (Supplementary Fig. 4b). Of note, clusters representing *Siah2*^*−/−*^ effector T cells (namely 7, 8, and 12) and the *Siah2*^*−/−*^ myeloid compartment (clusters 2, 6, 11, and 17 for macrophages, and clusters 14 and 18 for DCs) showed increased expression of genes implicated in glycolysis (Supplementary Fig. 4c, Supplementary Table 2), a metabolic switch observed in effector T cells^36^ and in activated DCs and proinflammatory macrophages^37^. These data confirmed that intratumoral immune cells in *Siah2*^*−/−*^ mice are more activated and proinflammatory. Analysis of number and frequency of cells in each cluster within the CD45^+^ (Ptrpc-expressing) cells obtained from WT and *Siah2*^*−/−*^ tumors revealed a decreased percentage of Tregs among intratumoral CD45^+^ cells obtained from *Siah2*^*−/−*^ relative to WT mice (Fig. 4b, Supplementary Table 5). No differences were observed in cluster 2, which represents most of the macrophage population. Yet, a notable decease seen in smaller macrophage clusters 6 and 17 (Fig. 4b, Supplementary Table 5) may represent a subgroup of macrophages that are either intrinsically affected by Siah2 deletion or by *Siah2*^*−/−*^ microenvironment. Overall, these findings substantiate the relative decrease in number of Foxp3^+^ cells in *Siah2*^*−/−*^ tumors seen in our FACS analysis.

The decreased frequency of tumor-infiltrating Tregs seen in *Siah2*^*−/−*^ mice could be attributed to alterations in the cell cycle, a possibility consistent with reports that *Siah2* loss in vitro decreases cell proliferation^24,26^. Single-cell RNAseq analysis of genes implicated in cell cycle control revealed inhibition of cell cycle progression in all *Siah2*^*−/−*^ immune cell clusters (Fig. 4c, Supplementary Fig. 4d). Notably, a subset of the CD8^+^ T cells (cluster 7) and Tregs (cluster 13) showed the most striking inhibition of the cell cycle in *Siah2*^*−/−*^ relative to WT genotypes (Fig. 4c, Supplementary Fig. 4e). The cell cycle phenotypes seen in tumor-infiltrating Tregs of *Siah2*^*−/−*^ mice coincided with an increased percentage of Treg cells in G1 of the cell cycle, indicating a G0/G1 block (Fig. 4c). Indeed, tumor-infiltrating Tregs from *Siah2*^*−/−*^ mice showed a striking decrease in proliferation (16% and 13% of them being in S and G2/M phases, respectively), compared to tumor-infiltrating Tregs in WT mice (32% and 26% in S and G2M, respectively). Accordingly, 71% of the Treg infiltrating to tumors in *Siah2*^*−/−*^ mice were found to be in G1/G0, compared with 42% infiltrated tumors in WT mice (Fig. 4c). To assess the rate of proliferation in each cluster, we analyzed Ki67 expression (Fig. 4d). The levels of Ki67 mRNA expression were highest in clusters 8 (containing CD8^+^ cells), 13 (Tregs), and 17 (a small cluster of macrophages) compared with other immune cell clusters (Fig. 4d). Notably, among these highly proliferative clusters, Tregs exhibited a significantly greater difference in expression of Ki67 between WT and *Siah2*^*−/−*^ genotypes (Fig. 4d). Further assessment of cell cycle-related genes identified decreased expression of E2F1 targets, including *Dnmt1*, within the Treg cluster (Fig. 4e, Supplementary Fig. 4f, Supplementary Table 3), a change observed in most immune cell clusters obtained from tumors grown in *Siah2*^*−/−*^ mice (Supplementary Fig. 4g).

To further establish changes in proliferation specific to Treg, we evaluated in vivo incorporation of bromodeoxyuridine (BrdU) in intratumoral immune cells. BrdU incorporation was traced in intratumoral T cells, but not in NK^+^ cells, macrophages or DCs. CD4^+^ conventional T cells and Tregs showed significantly decreased BrdU incorporation in *Siah2*^*−/−*^ compared to WT mice, whereas BrdU incorporation into CD8^+^ cells was comparable between genotypes (Fig. 4f). Importantly, decreased BrdU incorporation in CD4^+^ conventional T cells did not alter their frequency within tumors (Fig. 2a), as was seen for the Tregs. These observations mark Tregs as the primary immune cell type whose abundance was decreased in tumors from *Siah2*^*−/−*^ mice. The lack of decrease in number of tumor-infiltrating CD4^+^ T cells could be attributable to increased expression of *Cxcl9* that was observed in *Siah2*^*−/−*^ tumors (Fig. 1d), which could increase T cell recruitment, compensating for decreased CD4^+^ T cells proliferation. Indeed, immunohistochemical analysis revealed a significantly lower Ki67 expression in Foxp3^+^ cells from tumors grown in *Siah2*^*−/−*^ compared to WT mice (Fig. 4g). These observations substantiate the effect of Siah2 on Treg proliferation, resulting in their reduced abundance upon Siah2 loss.

### Treg proliferation is associated with p27 expression

**Fig. 5:**
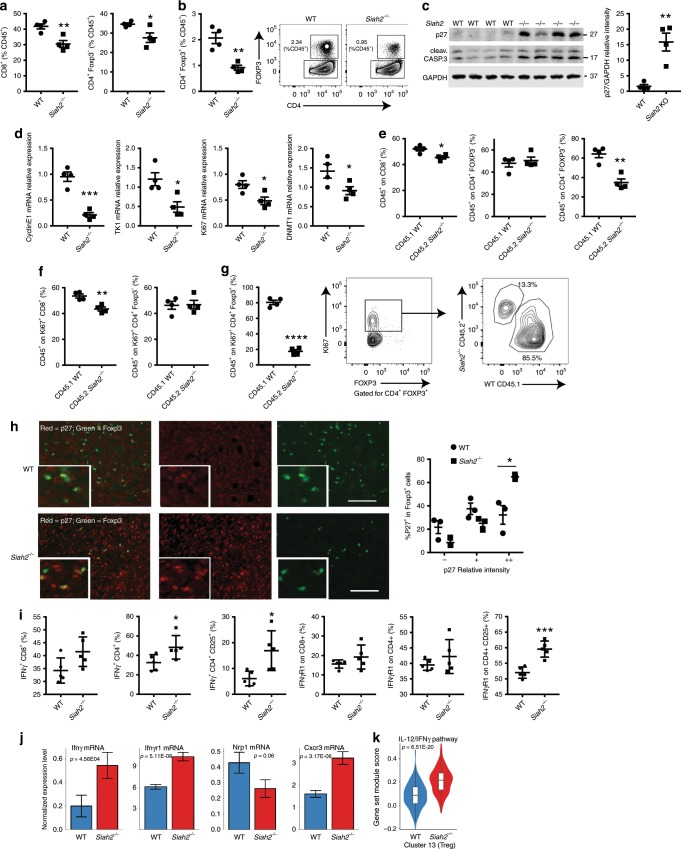
Increased p27, IFNγ, and IFNγR1 in intratumoral *Siah2*^*−*/*−*^ Tregs. **a**, **b** Lymphocytes from draining lymph nodes of *Siah2* WT and *Siah2*^*−/−*^ mice were cultured, stimulated with CD3/CD28 antibodies plus IL2, and 3 days later percentages of CD4^+^Foxp3^*−*^, CD4^+^Foxp3^+^, and CD8^+^ cells among CD45^+^ cells were determined by flow cytometry (*n* = 4). **c** Lysates from lymphocytes stimulated as in **a** were immunoblotted for p27 and cleaved caspase 3 plus GAPDH as a loading control (left; *n* = 4). Graph at right shows represents relative intensity of p27 protein between genotypes. **d** qPCR analysis of indicated transcripts from lymphocytes cultured as in **a** (*N* = 4, both genotypes). **e** T cells isolated from CD45.2^+^ *Siah2*^*−/−*^ spleen and CD45.1 WT spleen were cocultured (1:1), stimulated with CD3/CD28 antibodies plus IL2, and 3 days later percentages of CD45.1^+^ WT and CD45.2^+^ *Siah2*^*−/−*^ cells in CD4^+^Foxp3^*−*^, CD4^+^ Foxp3^+^, and CD8^+^ populations were determined by flow cytometry (*n* = 4). **f**, **g** The experiment was carried on as in **e**, and the percentage of CD45.1^+^ WT and CD45.2^+^ *Siah2*^*−/−*^ in Ki67^+^ CD4^+^Foxp3^+^ **g**, Ki67^+^CD4^+^ Foxp3^*−*^, and Ki67^+^CD8^+^ populations **f** was determined by flow cytometry (*n* = 4). **h** p27(red)/Foxp3(green) immunostaining (left panels) of tissues from tumors grown in *Siah2* WT and *Siah2*^*−/−*^ mice collected 11 days after melanoma cell injection, plus quantification (right; *n* = 3). Scale bar, 100 μm. **i** Frequencies of tumor-infiltrating IFN-γ^+^ or IFNγR1^+^ among CD8^+^, CD4^+^, and CD4^+^/CD25^+^ T cells 11 days after tumor inoculation and following overnight stimulation in vitro with PMA and Ionomycin (*n* = 5). **j** Expression of indicated mRNAs, as identified by single-cell RNAseq within the Treg cluster (cluster 13) in both genotypes. **k** Violin plot comparing expression levels of IL12/IFNγ-regulated genes, based on data obtained from single-cell RNAseq **k**; mean ± s.e.m. Data were analyzed by unpaired *t*-test in **a**–**i**, and by Wilcoxon rank-sum test in **j**–**k**. ***P < 0.0005, **P < 0.005, and *P < 0.05 compared with WT.

Siah2 activity has been often associated with cellular stress conditions (i.e., hypoxia, UPR). We thus assessed whether the effect of Siah2 on cell cycle requires T cell stimulation. In monitoring the percentages of T cell subpopulations, the proliferation of lymphocytes in draining lymph nodes from tumor-bearing *Siah2*^*−/−*^ or WT mice was assessed prior to and following stimulation in culture with CD3/CD28 antibodies combined with IL2. Prior to stimulation, a significant increase in the number of CD8^+^ cells and a decrease in the number of Foxp3^+^ cells in lymphocytes from *Siah2*^*−/−*^ compared to WT mice was noted (Supplementary Fig. 5a). Notably, after 3 days of stimulation in culture, a marked reduction in the number of Tregs obtained from *Siah2*^*−/−*^ compared to WT lymphocytes, relative to the changes seen in CD8^+^ and CD4^+^ FOXP3^−^ cells was observed (Fig. 5a, b). Flow cytometry analysis confirmed a significant decrease in Ki67 expression in CD8^+^, CD4^+^/FOXP3^−^, and CD4/FOXP3^+^ populations in *Siah2*^*−/−*^ relative to WT mice (Supplementary Fig. 5b), confirming changes in cell proliferation.

Among cell cycle regulatory proteins reportedly controlled by Siah2 during T cell activation is the cyclin-dependent kinase inhibitor p27^24,26^. Analysis of p27 expression in unstimulated lymphocytes derived from *Siah2*^*−/−*^ or WT draining lymph nodes revealed comparable expression (Supplementary Fig. 5c). However, stimulated lymphocytes from *Siah2*^*−/−*^ draining lymph nodes showed a marked (15-fold) increase in p27 protein levels relative to lymphocytes from WT mice (Fig. 5c), and a moderate induction of p27 mRNA relative to WT lymphocytes (Supplementary Fig. 5d). An increase in expression of cleaved caspase 3 protein (Fig. 5c) was also noted in activated lymphocytes from *Siah2*^*−/−*^ mice (compared to those obtained from WT mice), suggesting that p27 upregulation also promoted apoptosis^38,39^.

Given that p27 induces G1 block in T cells^40,41^ and reduces E2F1 transcriptional activity^42^, we analyzed changes in transcription of E2F1 target genes in in vitro stimulated *Siah2*^*−/−*^ and WT lymphocytes. Indeed, decreased expression of the E2F1 targets *Cyclin E1, Tk1, Ki67, and Dnmt1* in *Siah2*^*−/−*^ activated lymphocytes compared to WT cells was observed (Fig. 5d). Reduced levels of E2F1 target genes in the intratumoral Treg cluster, as identified by single-cell RNAseq were also noted (Fig. 4c). Impaired proliferation of *Siah2*^*−/−*^ Treg cells could be due to either an intrinsic proliferation defect or a response to cytokines or growth factors secreted during activation. To distinguish between these possibilities, T cells were isolated from spleens of CD45.1^+^ WT mice, mixed (1:1) with T cells from spleens of CD45.2^+^ *Siah2*^*−/−*^ mice, and then stimulated (with CD3/CD28 plus IL-2) for 3 days, followed by FACS analysis. Notably, a lower (35%) percentage of CD45.2^+^ *Siah2*^*−/−*^ Tregs compared to 65% of CD45.1^+^ WT Treg cells was found within the FOXP3^+^/CD4^+^ cell population. A small but statistically significant decrease in frequency of CD45.2^+^ *Siah2*^*−/−*^/CD8^+^ cells was also noted among the CD8^+^ population, but not in CD45.2^+^ *Siah2*^*−/−*^ CD4^+^ FOXP3^−^ cells (Fig. 5e) compared to CD45.1^+^ cells. In agreement, a lower percentage (20%) of CD45.2^+^ *Siah2*^*−/−*^ cells was seen within the Ki67^+^FOXP3^+^CD4^+^ cell population (Tregs) compared with 80% of CD45.1^+^ WT cells (Fig. 5g). In these experiments, the frequency of CD45.2^+^ *Siah2*^*−/−*^ and CD45.1^+^ WT cells, was largely comparable within Ki67^+^ nonTreg populations, including the Ki67^+^/CD8^+^ and Ki67^+^/CD4^+^/FOXP3^*−*^ populations, although a modest, albeit significant, decrease in frequency of CD45.2 *Siah2*^*−/−*^ cells was observed in the Ki67^+^ CD8^+^ population (Fig. 5f). Collectively, these findings suggest an intrinsic cell proliferation defect in T cells of *Siah2*^*−/−*^ mice, in which the Treg population is the most impacted. Immunohistochemistry analysis showed a significant increase of p27^+^FOXP3^+^ cells (Fig. 5h) in YUMMER1.7 melanoma tumors grown in *Siah2*^*−/−*^ compared to WT mice, confirming increases in levels of p27 protein in the *Siah2*^*−/−*^-derived intratumoral Treg cell population and strongly suggesting that Siah2 controls Treg proliferation via p27.

To assess the possibility that changes in Siah2 activity alter Treg immunosuppressive capacity, we performed an in vitro suppression assay using WT and *Siah2*^*−/−*^ Tregs. In culture, Treg capacity to suppress CD4^+^ cell proliferation did not differ between genotypes (Supplementary Fig. 5e). Correspondingly, intratumoral Tregs did not exhibit differences in TGFβ or IL10 production between genotypes (Supplementary Fig. 5f). Suppressive function of Tregs assessed in vivo was previously shown to differ from that assessed in culture independent of *Foxp3* expression, while lack of immune suppressive function has been linked to Treg expression of IFNγ and its receptor^43,44^. Consistent with earlier studies, showing induction of IFNγ expression upon reduced DNMT1 expression in Tregs grown in vivo^45^, we identified reduced expression of the E2F1 target gene *Dnmt1* expression (Supplementary Fig. 4f) and a notable increase in IFNγ and IFNγ receptor (IFNγR1) levels in intratumoral Tregs from *Siah2*^*−/−*^ compared to WT mice (Fig. 5i). Intratumoral CD8^+^ and CD4^+^ cells exhibited increased IFNγ levels and comparable levels of its receptor IFNγR1 in *Siah2*^*−/−*^ (Fig. 5i) relative to corresponding WT cells. These findings were supported by single-cell RNAseq analysis, which confirmed increased expression of *Ifnγ* and *Ifnγr1* within the Treg cluster, and decreased *Nrp1* expression (Fig. 5j). Of note, as reported for intratumoral *Nrp1*^*−/−*^ Tregs that exhibit a Treg fragility phenotype^44^ the *Siah2*^*−/−*^ Treg cluster exhibited increased expression of the chemokine receptor *Cxcr3*, a marker of type 1 helper T cells (Fig. 5j, Supplementary Fig. 5g), and increased expression of genes associated with the IL12/ IFNγ pathway (Fig. 5k, Supplementary Table 4). Increased expression of the chemokine receptor CCR8^46,47^, along with decreased expression of its ligand *Ccl1* was seen in Tregs infiltrating to tumors grown in *Siah2*^*−/−*^, compared with WT mice (Supplementary Fig. 5g).

Given the decrease in Treg cell number observed in tumors grown in *Siah2*^*−/−*^ mice, the basal Treg frequency was assessed. Treg population in tumor-free *Siah2*^*−/−*^ mice revealed a mild, albeit significant, reduction in Treg number (by 20%) in lymph nodes and spleen (Supplementary Fig. 5h, i), prompting the assessment of possible changes in T cell populations in thymus. While the percentage of immature CD4^+^/CD8^+^ double-positive and CD4^+^ or CD8^+^ single-positive populations in *Siah2*^*−/−*^ mouse thymus remained largely unchanged (Supplementary Fig. 5j), a small yet significant decrease in the number of CD4^+^Foxp3^+^ cells and in Treg precursors (CD4^+^ CD25^+^ GITR^+^ Foxp3^*−*^ cells) in thymus of *Siah2*^*−/−*^ relative to WT mice was seen (Supplementary Fig. 5k, l). Notably, no signs of autoimmunity were seen in *Siah2*^*−/−*^ mice, up to 1 year of age.

### Inhibition of proliferation in *Siah2*^*−*/*−*^ cells is p27 dependent

**Fig. 6:**
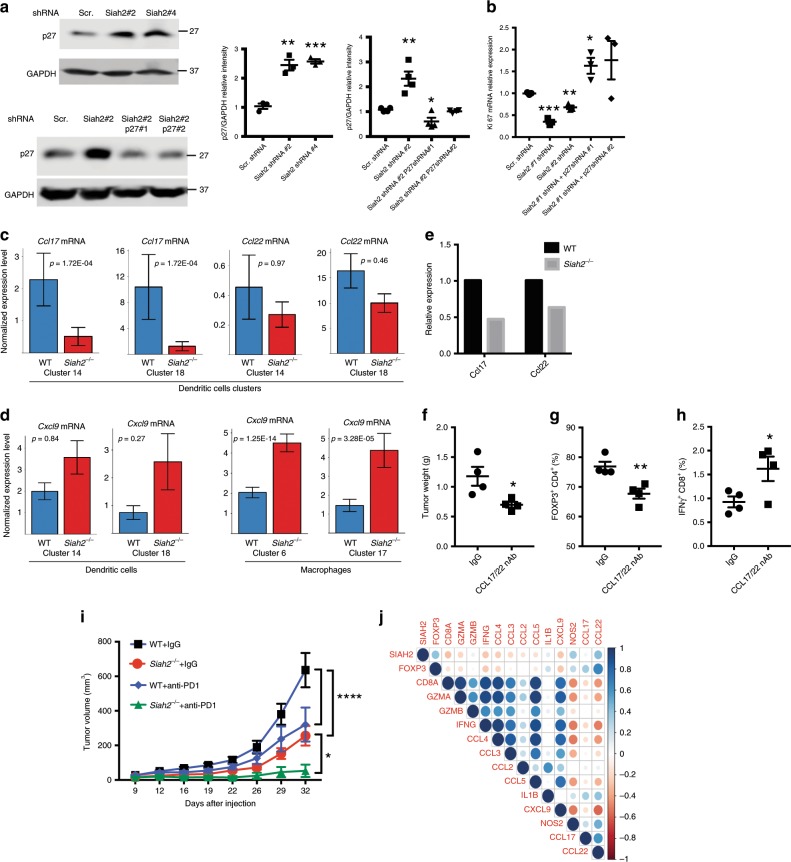
*Siah2*^*−/−*^ effect on cell proliferation is p27-dependent **a**, **b** Jurkat cells were depleted of Siah2 alone or of Siah2 and p27 via infection with lentivirus harboring indicated shRNAs. Proteins prepared were analyzed by immunoblot for p27 and GAPDH as loading control (*n* = 4; relative intensity of p27 shown in the graphs) **a**, while RNA prepared was processed for qPCR analysis of *Ki67* transcripts **b**. **c** *Ccl17* and *Ccl22* mRNA expression, as identified by single-cell RNAseq within dendritic cell clusters (C14 and C18) in both genotypes. **d** *Cxcl9* mRNA expression, as identified by single-cell RNAseq within dendritic cell clusters (C14 and C18) in both genotypes. **e** *Ccl17* and *Ccl22* mRNA expression of CD11c^+^-sorted cells from tumors from both genotypes. Ten tumors were collected per sample 11 days after YUMMER1.7 cell inoculation. Data are representative of two independent experiments. **f**–**h** Weight of tumors collected 19 days after YUMMER1.7 cell inoculation of WT mice, which were injected i.p. with CCL17 and CCL22 neutralizing antibodies every other day, starting 3 days after melanoma cell inoculation (*n* = 4) **f**. At the end of the treatment described in **f** (day 19), tumors were collected and frequencies of tumor-infiltrating Foxp3^+^ cells within the CD4^+^ T cell population **g** and of IFN-γ- expressing CD8^+^ cells (*n* = 4) were determined **h**. **i** Loss of Siah2 synergizes with PD1 therapy. Mean growth curves over time of tumors derived from YUMM1.7 cells (150,000) injected into WT and *Siah2*^*−/−*^ mice, which were then treated with anti-PD-1 antibody (200 μg/mouse; three times per week for a total of five times) or rat isotype (IgG) starting at day 7 after melanoma cell injection. WT and *Siah2*^*−/−*^ IgG (*n* = 8); WT and *Siah2*^*−/−*^ anti*-*PD-1 antibodies (*n* = 7). Shown are complete regression (CR) rates at study termination. **j** Positive correlation between Siah2 and Foxp3 expression in immunogenic melanoma tumors. Spearman’s rank correlation plots (scatterplots) for pairwise comparisons between *SIAH2* expression (mRNA *z*-scores) and the Genset identified in Treg immune signature seen in *Siah2*^*−/−*^ mice expressed in the metastatic samples with high immune signals from TCGA_SKCM (*n* = 66)^48^; mean ± s.e.m. Data were analyzed by Wilcoxon rank-sum test **c**, **d**, unpaired *t*-test **a**, **b**, **f**–**h** or two-way ANOVA with Bonferroni multiple comparison **i**. ***P < 0.0005, **P < 0.005, and *P < 0.05.

To assess whether increased p27 protein levels observed in *Siah2*^*−/−*^ cells inhibit proliferation, changes in Ki67 expression in cultured Jurkat T cells following knockdown of Siah2 with or without p27 knockdown were monitored (Supplementary Fig. 6a, b). While Siah2 knockdown alone significantly increased p27 protein levels and decreased *Ki67* expression (Fig. 6a, b), concomitant p27 knockdown rescued *Ki67* expression to WT levels (Fig. 6a, b). Furthermore, *Siah2*^*−/−*^ Tregs isolated from spleen and expanded in vitro showed decreased *Ki67* expression relative to Treg derived from WT mice, a decrease reversed by p27 knockdown (Supplementary Fig. 6c, d). These data suggest that decreased cell proliferation seen upon Siah2 knockdown or deletion is p27 dependent.

### Lower intratumoral Ccl17/22 expression in tumor inhibition

Both RNAseq and NanoString analyses showed that melanoma tumors grown in *Siah2*^*−/−*^ mice exhibit reduced expression of *Ccl17* and *Ccl22*, chemokines expressed by tolerogenic DCs^49,50^, along with increased expression of *Cxcl9*, a chemokine functioning in T cell recruitment to tumor sites (Fig. 1d). These observations were confirmed by single-cell RNAseq analysis that revealed increased *Cxcl9* expression in the *Siah2*^*−/−*^ myeloid compartment, and identified *Ccl17* and *Ccl22* expression in the DC clusters (14 and 18; Fig. 6c, d). Indeed, a significant decrease in *Ccl17* and *Ccl22* mRNA levels was confirmed in CD11c^+^ cells sorted from *Siah2*^*−/−*^-derived tumors (Fig. 6e). To determine whether those decreases underlie inhibition of tumor growth, Siah2 WT mice were treated with CCL17- and CCL22 neutralizing or control antibodies, 2 days after inoculating animals with YUMMER1.7 melanoma cells. Notably, administration of CCL17 and CCL22 antibodies led to a 30% inhibition in tumor weight relative to treatment with control antibody (Fig. 6f). Treg infiltration of the tumor site decreased by ~25% (Fig. 6g), concomitant with a 50% increase in IFNγ expression within the CD8^+^-positive population (Fig. 6h). These data suggest that CCL17 and CCl22 mediate, in part, tumor inhibition phenotypes seen in *Siah2*^*−/−*^ mice.

### Siah2 loss synergizes with effects of anti-PD1 therapy

Phenotypes observed in intratumoral immune cells in tumors grown in *Siah2*^*−/−*^ mice mirror those reported for tumor-infiltrating immune cells following treatment with CDK4/6 inhibitors^51–53^. Since treatment with CDK4/6 inhibitors potentiates anti-PD-1 ICT therapy, we asked whether *Siah2*^*−/−*^ loss would alter the effectiveness of anti-PD-1 ICT in mice. WT and *Siah2*^*−/−*^ mice were inoculated with YUMM1.7 cells and 7 days later both groups were treated with anti-PD-1 antibody. YUMM1.7 cells were selected since they exhibit a limited tumor inhibition following treatment with anti-PD1 antibodies^32^. Notably, *Siah2*^*−/−*^ mice not treated with anti-PD-1 antibody exhibited decreased tumor growth, with one of eight tumors regressed, which was not seen in the WT background. Significantly, treatment of *Siah2*^*−/−*^ mice with anti-PD1 antibodies promoted tumor regression in five of seven animals (Fig. 6i, Supplementary Fig. 6e), while only one of seven WT mice exhibited tumor regression in response to PD-1 blockade (Fig. 6i, Supplementary Fig. 6e). Similarly, growth of MC38 colon cancer cells in *Siah2*^*−/−*^ mice was attenuated following administration of anti-PD-1 antibodies compared with comparable treatment of WT mice (Supplementary Fig. 6f). These findings demonstrate that anti-PD-1 therapy synergizes with loss of Siah2 and promotes tumor regression.

### Siah2-Foxp3 expression correlates in immunogenic specimens

The clinical relevance of our findings was assessed by monitoring changes in Siah2 expression in the TCGA skin melanoma cohort (TCGA_SKCM). The signature of genes that were differentially expressed in the *Siah2*^*−/−*^ grown tumors was compared with those grown in the WT mice (Fig. 1c). To focus the analysis on immune infiltrated metastatic melanomas, the TCGA cohort (*n* = 339) was stratified for “low”, “intermediate”, or “high” immune signature expression^48^. The analysis of the high immune responsive subset (*n* = 66) identified a positive correlation between the expression of *FOXP3, CCL22,* and *SIAH2* (*r* = 0.4, *P* = 0.001; *r* = 0.4, *P* = 0.03, respectively) and a negative correlation between the expression of *SIAH2* and of *CD8α, IFN*γ, and *CXCL9* (*r* = −0.3, *P* = 0.03; *P* = −0.3, *P* = 0.05; *r* = −0.3, *P* = 0.04, respectively; Fig. 6j and Supplementary Fig. 6G). A positive correlation between *SIAH2* and *FOXP3* (Tregs marker) expression suggests that findings derived from a genetic murine melanoma model are relevant to human tumors and that low Siah2 levels could serve as a marker to stratify patients for ICT therapy.

## Discussion

Here, we identify a role for the ubiquitin ligase Siah2 in control of intratumoral Treg proliferation and tumor infiltration. Relative to WT mice, *Siah2*^*−/−*^ mice inoculated with YUMMER1.7 melanoma cells showed a decreased number of Tregs within tumors as well as inhibited tumor growth, up to complete regression. Our data points to changes in Treg proliferation, suppressive function and recruitment which collectively impact their abundance in tumors, enabling enhanced anti-tumor immunity.

Siah2-dependent cell autonomous regulation of Tregs with concomitant effect on immune cell activation is supported by: (i) Single-cell RNAseq performed on immune cells infiltrating tumors obtained from *Siah2*^*−/−*^ or WT mice showed that cells in the Treg cluster (#13) exhibited the most striking G1 block in *Siah2*^*−/−*^ relative to WT genotypes. (ii) Analysis of Ki67 expression across distinct immune populations from *Siah2*^*−/−*^ or WT mice confirmed that the Treg population exhibited the greatest proliferative difference between genotypes. (iii) BrdU incorporation assays performed in vivo identified a significant decrease in BrdU incorporation in Treg cells. (iv) A mixed BM chimera analysis demonstrated that decreased cell number seen in Tregs of *Siah2*^*−/−*^ mice is due to cell-intrinsic mechanisms rather than to activities of other immune cells.

Among immune cells that infiltrate tumors, T cells are the most proliferative and are thus more susceptible to Siah2-dependent control of cell cycle progression. Intratumoral *Siah2*^*−/−*^ T cells exhibited increased effector functions (among them, increased expression of IFNγ, TNFα, and granzyme B), increased expression of NFAT/cell identity genes (CD3δ, CD3ε, and CD3γ), and decreased Treg frequency. Since Siah2 loss impacts effector T cell, we set to clarify the nature of the differential changes observed for Tregs, in vivo. Lack of nutrients and low oxygen, both commonly seen in the tumor microenvironment, limit proliferation of most immune cells that depend on glycolytic metabolism. Yet, Tregs rely mostly on oxidative phosphorylation, which provides them with a proliferative advantage^29,30^ and may render them more responsive to *Siah2* deletion. Thus, among the diverse T cell populations, the intratumoral Tregs are the most sensitive to *Siah2* deletion, reflected by the highest degree of G1 block among tumor-infiltrated immune cell populations. While changes in cell cycle control  were previously seen in Siah2^*−*/*−*^ derived mouse embryonic fibroblasts^54^, neither their effect on  T cell subpopulations nor the mechanism underlying these changes were reported. Although we did not observe defects in Treg immunosuppressive capacity in *Siah2*^*−/−*^ Tregs in vitro, changes identified in intratumoral *Siah2*^*−/−*^ Tregs in vivo, suggest impaired immunosuppressive function in these cell population. The latter is supported by noting increased expression of both IFNγ and IFNγr1, and decreased Nrp1 expression in intratumoral Tregs of *Siah2*^*−/−*^ mice, consistent with decreased Treg immunosuppressive function in vivo previously reported^44^. Thus, immunosuppressive phenotypes may be apparent only in the context of the tumor environment. Consistent with the preferred effect on tumor-infiltrating Treg is the observation that *Siah2*^*−/−*^ mice did not exhibit autoimmune or other immune-related disorders (our unpublished data).

We also show changes in DCs, where Siah2 loss increased expression of the IFNγ-induced chemokine *Cxcl9*, which recruits effector T cells to a tumor site, and concomitantly decreased expression of *Ccl17* and *Ccl22*, chemokines that contribute to recruitment of Tregs to tumors. Notably, cBioPortal analysis of TCGA data relevant to *CCL22* expression in melanoma patients revealed a strong positive correlation between *CCL22* and *FOXP3* mRNA expression (Pearson = 0.66, Spearman = 0.77, *P* = 0.02). Furthermore, levels of *CCL17* mRNA correlated positively with levels of *CCL22* mRNA expression (Pearson = 0.62, *P* = 0.06), findings that link our observations in *Siah2*^*−/−*^ mice to clinical outcomes seen in melanoma patients. Our ability to rescue, in part, Treg recruitment and tumor growth in *Siah2*^*−/−*^ mice subjected to treatment with neutralizing antibodies to *Ccl17* and *Ccl22* further illustrates the independent pathways by which Siah2 controls tumor growth. Additional evidence for the physiological significance of our findings comes from analysis of human melanoma tumors in the TCGA dataset. A significant positive correlation between expression of *SIAH2* and the immune gene signature of tumors grown in our *Siah2*^*−/−*^ mouse model signified the immune-responsive human melanoma tumors. The latter substantiates the significance of our finding in murine melanoma models and suggests potential use of Siah2 expression as a marker to stratify melanoma cases for ICT.

The finding that reduced proliferation of T cells can be attributed to Siah2 regulation of p27 stability is consistent with previous reports^24,26^. Siah2 knockdown in the Jurkat T cells increased p27 protein levels and decreased proliferation, changes reversed by co-deletion of p27. Siah2-dependent effects on p27 are likely manifested following the stress accompanying immune cell stimulation (via T cell receptor signaling) or harsh tumor environment, conditions implicated for Siah2 activities^55^. Cell cycle arrest phenotypes seen following Siah2 loss resemble those observed following treatment with CDK4/6 inhibitors^51–53^. Indeed, decreased proliferation and cell cycle arrest seen in single-cell-based RNAseq analysis of tumor-infiltrated T cells in *Siah2*^*−/−*^ mice resemble changes observed following use of pharmacological CDK4/6 inhibitors currently in clinical trials^52^. Common to both is a robust increase in anti-tumor immunity. Administration of Siah2 inhibitors could thus provide a therapeutic advantage in modulating cell cycle progression in a select immune cell population, which we show to be Tregs, and represent a novel therapeutic modality. Although the development of Siah2 inhibitors has been a challenging task, our findings justify hastening those efforts.

In all, we demonstrate the ability to target tumors that are nonresponsive to PD-1 therapy by providing an environment limiting Treg suppressive function, as observed in the *Siah2*^*−/−*^ mice. Limiting Treg infiltration or suppressive function is also expected to support CTLA4-based therapy, which does not impact this select immune cell population^56^. Our data further supports the notion that combining ICT with cell cycle-disrupting drugs provides an effective strategy to promote an anti-tumor response. Targeting the ubiquitin ligase Siah2, central in cell autonomous proliferation and activity of Tregs, may thus offer the rationale for an innovative therapeutic approach.

## Methods

### Animals and tumor models

All experimental animal procedures were approved by the Institutional Animal Care and Use Committee of Sanford Burnham Prebys Medical Discovery Institute (approval # 16–070, 17–043) and complied with all relevant ethical regulations for animal testing and research. *Siah2*^*−/−*^ mice were generated as previously described^54^. *Braf*^*V600E/+*^;* Pten*^*−/−*^; and *Cdkn2a*^*−/−*^ mouse melanoma cells (YUMM1.7, YUMMER1.7) were kindly provided by Marcus Bosenberg^31,32^. For tumor growth experiments, mice were injected s.c. with 400,000 YUMMER1.7 cells or 150,000 YUMM1.7 cells, or 500,000 MC38 cells, unless otherwise noted. Tumor volumes were measured twice a week. Tumors were collected 10–14 days after inoculation, unless otherwise noted.

### BM chimeras

WT or *Siah2*^*−/−*^ recipient mice were lethally irradiated (1000 rads) and reconstituted by intravenous (i.v.) injection of 1 × 10^7^ BM cells isolated from femurs and tibias of donor WT or *Siah2*^*−/−*^ mice. Recipients were treated with antibiotics (trimethoprim 8 mg/ml and sulfamethoxazole 40 mg/ml in drinking water) for 3 weeks after injection. Reconstitution was confirmed 6–8 weeks after BM transfer, and chimeric mice were then injected s.c. with 400,000 YUMMER1.7 cells. For mixed BM chimeras, experiments were carried out as for BM chimeras, except that irradiated mice were injected i.v. with a 1:1 mixture of *Siah2* WT Thy1.1 and *Siah2*^*−/−*^ Thy1.2 cells, for a total 1 × 10^7^ BM cells.

### Tumor digestion

Tumors were excised, minced, and digested with 1 mg/ml collagenase D (Roche) and 100 µg/ml DNase I (Sigma) at 37 °C for 1 h. Digests were then passed through a 70-μm cell strainer to generate a single-cell suspension. Cells were washed twice with phosphate-buffered saline (PBS) containing 2 mM ethylenediaminetetraacetic acid (EDTA) and stained for flow cytometry.

### Flow cytometry

Tumor-derived single-cell suspensions were washed twice with FACS staining buffer, fixed 15 min with 1% formaldehyde in PBS, washed twice, and resuspended in FACS staining buffer. For intracellular cytokine staining, cells were resuspended in complete RPMI-1640 (containing 10 mM HEPES, 1% nonessential amino acids and L-glutamine, 1 mM sodium pyruvate, 10% heat-inactivated fetal bovine serum (FBS), and antibiotics) supplemented with 50 U/mL IL-2 (NCI) and 1 mg/mL brefeldin A (BFA, Sigma), and then incubated either with phorbol myristate acetate (10 ng/ml) and ionomycin (0.5 μg/ml) or with melanoma peptides made by Anaspec Inc at 2 μg/ml: MGP100_25–33_ (AS-64752); melan-A_26–35_, (AS-61011); TRP2_180–188_, (AS-61058); and TRP2_181–188_ (AS-64811) for 16 h at 37 °C. Cells were then fixed and permeabilized using a Cytofix/Cytoperm Kit (BD Biosciences) before staining.

Antibodies were purchased against the following proteins: CD45.2 (104, 1:200), CD8α (53–6.7, 1:200), CD4 (GK1.5, 1:200), CD45.1 (A20, 1:200), TNFα (MP6-XT22, 1:100), IFNγ (XMG1.2, 1:100), CD11c (N418, 1:200), CD11b (M1/70, 1:200), MHC class I (AF6–88.5, 1:200), CD80 (16-10A1, 1:200, 1:200), FOXP3 (FJK-16s, 1:100), CXCR3 (CXCR3-173), CCR8 (SA214G2, 1:200), GR1 (RB6-8C5, 1:200), CD206 (C068C2, 1:200), Thy1.1 (OX-7, 1:200), Thy1.2 (53-21, 1:200), KI67 (16A8, 1:100), TGFβ (TW7-16B4, 1:100), IL10 (JES516E3, 1:100), GITR (DTA-1, 1:200), TBET (4B10, 1:100), and CD25 (3C7, 1:200) were purchased from BioLegend, while GRANZYME B (GB11, 1:10) and NOS2 (CXNFT, 1:100) were purchased from BD Biosciences, and IFNγr1 was purchased by Miltenyi Biotec Inc (130104934, 1:100). All data were collected on an LSRFortessa cell analyzer (BD Biosciences) and analyzed using FlowJo Software (Tree Star). Gating strategy is provided in Supplementary Fig. 7.

### Histology and immunofluorescence

Tumors collected 11 days after melanoma cell injection were fixed in 4% formalin overnight at 4 °C, washed with PBS, paraffin-embedded, cut into 5 μm-thick sections and stained with hematoxylin and eosin. For immunofluorescence, sections were deparaffinized, rehydrated, and washed in PBS. Antigen retrieval was performed in a pressure cooker (Decloaking chamber, Biocare Medical) in citrate buffer (pH 6.0). Foxp3 (ThermoFisher LifeTechnology FJK-165, 1:100), Ki67 (AbCam Ab15580, 1:250), CD3 (Novus Biologicals NB600-144, 1:50), and p27 (Santa Cruz; C-19, 1:25) immunostaining was performed by incubating sections overnight at 4 °C with antibodies in Dako antibody diluent. Alexa Fluor 488- or Alexa Fluor 594-conjugated secondary antibodies were added for 1 h at room temperature (Molecular Probes), and nuclei were counterstained using SlowFade Gold Antifade reagent (Vector) with 4′,6-diamidino-2-phenylindole (DAPI, Vector).

Image data were obtained using an Olympus TH4–100 microscope and Slidebook 4.1 digital microscopy. For quantification, Ki67-, Foxp3-, CD3-, and p27-positive cells were counted in five random ×20 fields per mouse. Staining was scored using a three-tiered intensity scale ranging from 0 (no staining) to + + (highest intensity).

### Cell lines and gene silencing

YUMM1.7 and YUMMER1.7 cells were kindly provided by Marcus Bosenberg^31,32^ and grown in DMEM media supplemented with 10% FBS and Amp/Pen. MC38 cell line was obtained from Kerafast and was grown in DMEM media supplemented with 10% FBS and Amp/Pen. Jurkat cells were obtained from ATCC and grown in RPMI supplemented with 10% FBS and Amp/Pen. Short hairpin RNAs (shRNAs) were purchased from Sigma-Aldrich (human Siah2 #2 TRCN0000297333; human Siah2#4 TRCN0000297339; human p27#1 TRCN0000039928; human p27#3 TRCN0000039932; mouse p27#1 TRCN0000294885; mouse p27#2 TRCN0000287390; non targeted shRNA control; SHC016). Lentiviral particles were prepared using standard protocols. Briefly, HEK293T cells obtained from ATCC were transfected with shRNA plasmid and the second-generation packaging plasmids delta R8.2 and VSV-G (Addgene). Viral supernatants were collected 48 h later and used in the presence of polybrene (Sigma) to infect indicated lines. Cell lines were authenticated at SBP Genomic Core using short tandem repeat (STR) analysis which was performed on isolated genomic DNA with the GenePrint® 10 System from Promega, and peaks were analyzed using GeneMarker HID from Softgenetics. Allele calls were searched against STR databases maintained by ATCC (www.atcc.org), DSMZ (www.dsmz.de), Texas Tech University Children’s Oncology Group (cogcell.org), and the Wistar Institute Melanoma Cell STR Profiles (http://www.wistar.org/lab/meenhard-herlyn-dvm-dsc/page/melanoma-cell-str-profiles). All cell lines were maintained under mycoplasma-free conditions.

### Western blotting

Cells were washed once with PBS at room temperature and resuspended in RIPA buffer (PBS containing 1% NP-40, 1% sodium deoxycholate, 1% sodium dodecyl sulfate (SDS), 1 mM EDTA, and phosphatase and protease inhibitors), while tissue was homogenized directly in RIPA buffer. Lysates were centrifuged, and supernatants were removed and subjected to SDS–polyacrylamide gel electrophoresis. Proteins were transferred to nitrocellulose membranes (Osmonics Inc., MN, USA), which were blocked and incubated with respective primary antibodies followed by Alexa Fluor-conjugated secondary antibodies. The first antibodies p27 (3688, 1:1000), GAPDH (5174, 1:1000), and cleaved caspase 3 (9664, 1:1000) were all purchased from Cell Signaling. Blots were imaged using an Odyssey detection system (Amersham Bioscience, NJ, USA). Uncropped blots provided in Source Data File.

### BrdU incorporation

BrdU incorporation was performed following a protocol from the FITC-BrdU kit (BD Pharmingen). Briefly, mice were injected intraperitoneally (i.p.) with 1 mg BrdU solution and sacrificed 20 h later. Tumors were digested and cells stained for surface markers. Cells were fixed and permeabilized with BD cytofix/cytoperm buffer, followed by DNAse I digestion, and stained with anti-BrdU antibody.

### In vivo antibody treatments

Anti-CCL17 and anti-CCL22 neutralizing antibodies were purchased (R&D systems). Mice were injected with both antibodies (20 μg/dose dissolved in 200 μl sterile normal saline), starting 2 days after tumor injection, and every other day thereafter until tumor collection. For anti-PD-1 antibody treatment, mice were injected (i.p.) with 200 μg anti-PD-1 clone RMP1-14 for YUMM1.7, and 100 μg anti-PD-1 clone RMP1-14 for MC38 cells, or rat IgG2a isotype control on days 7, 9, 12, 14, and 16 after inoculation of YUMM1.7 cells, and on days 7, 9, 12, and 14 after inoculation of MC38 cells.

### RNA extraction and qRT-PCR analyses

Total RNA was extracted from tumors or cells using TRIzol (Ambion) and treated with DNase I. cDNA was synthesized using oligo-dT and random hexamer primers according to the SYBR Green qPCR protocol (Life Technologies). Total RNA was reverse transcribed using high Capacity Reverse Transcriptase kits (Invitrogen), according the manufacturer’s protocol. Purity and concentration of extracted RNA were checked and quantified by reading absorbance at 260 and 280 nm in a NanoDrop spectrophotometer (Thermo Fisher). Quantitative real-time PCR (qRT-PCR) analyses were performed using SYBR Green RT-PCR kits (Invitrogen) on a Bio-Rad CFX Connect Real-Time system or Roche LightCycler. GAPDH or 18 S was amplified as an internal control. PCR primers were designed using Primer3, and their specificity was checked using BLAST. PCR products were limited to 100–200 bp. Primer sequences are shown in Supplemental Table 1.

### NanoString nCounter assay

For each assay, a 100 ng aliquot of RNA was mixed with a NanoString code set mix and incubated at 65 °C overnight (16 h). Reaction mixes were loaded onto the NanoString nCounter Prep Station for binding and washing, and the resulting cartridge was transferred to the NanoString nCounter digital analyzer for scanning and data collection. Quantified expression data were analyzed using NanoString nSolver Analysis Software v2.0. After performing image quality control using a predefined cutoff value, we excluded outlier samples using a normalized factor based on the sum of positive control counts >3-fold. Data were normalized by scaling with the geometric mean of built-in control gene probes for each sample. Data tabulated in the heatmap were based on 364 DEGs (comparing Siah2 KO to WT), of which 87 genes were downregulated and 277 were upregulated, with *P* < 0.05. NanoString data has been deposited in public dataset: GSE 134328.

### Bioinformatics and statistical analysis of the NanoString nCounter assay

For gene expression data from the NanoString nCounter assay, filtering of samples using quality control criteria was performed according to the manufacturer’s recommendations. Raw counts of samples passing quality control were normalized using 20 reference genes (Abcf1, Alas1, Edc3, Eef1g, Eif2b4, G6pdx, Gusb, Hdac3, Hprt, Nubp1, Oaz1, Polr1b, Polr2a, Ppia, Rpl19, Sap130, Sdha, Sf3a3, Tbp, and Tubb5) as internal controls. Data were log2-transformed and further analyzed. Student’s *t*-test was applied to compare normalized expression values between groups. IPA (Ingenuity Pathway Analysis) analysis for the 364 differentially expressed genes (DEGs) was used to map biological processes, pathways, and networks.

### RNAseq analysis

PolyA RNA was isolated using the NEBNext® Poly(A) mRNA Magnetic Isolation Module, and bar-coded libraries were constructed using the NEBNext® Ultra™ Directional RNA Library Prep Kit for Illumina® (NEB, Ipswich MA). Libraries were pooled and single end sequenced (1 × 75) on the Illumina NextSeq 500 using the High output V2 kit (Illumina Inc., San Diego CA). Raw data QC by FASTQC (https://www.bioinformatics.babraham.ac.uk/projects/fastqc/) and mapping to the mouse reference genome (mm10) by STAR^57^ with default parameters were performed in Illumina BaseSpace (https://basespace.illumina.com). FeatureCounts^58^ was used to count reads mapped to the annotated mouse genes. The EdgeR-based R pipeline in SARTools^59^ was used to identify DEGs. DEGs with *P* < 0.01 and |log2Foldchange| > 2 are shown in the heatmap, and DEGs with *P* < 0.01 and |log2Foldchange| > 1 are included in IPA pathway analysis. Data obtained under this analysis was deposited in public dataset: GSE134412.

### Single-cell library preparation and sequencing

*Siah2*^*−/−*^ and WT mice were sacrificed 11 days after tumor cell inoculation. Tumors from each group were minced prior to incubation with 0.3 Wünsch U/mL Liberase TM (Sigma) and 50 U/ml Dnase I (Roche) in Hank’s Balanced Salt Solution (Life Technologies) for 30 min at 37 °C with agitation. Tumors were homogenized by repeated pipetting and filtered through a 70-μm nylon filter. Single cell suspensions were washed with 1 × PBS-4%FBS before incubation 20 min on ice at 5 × 10^7^ cells/ml with 500 ng/ml Fc block (2.4G2, BD Pharmingen) to prevent nonspecific antibody binding. Cells were then incubated 1 h on ice with PE-eFluor610-conjugated CD45 monoclonal antibody (30-F11, eBioscience). For scRNAseq libraries, DAPI-negative (live) CD45^+^ and CD45^−^ cells were sorted using a SY3200 flow cytometer and sorted cells were resuspended in RPMI for counting. Live CD45^+^ and CD45^−^ cells were mixed 5:1. Libraries were prepared using a Single Cell 3′ Reagent Kit v2, a Chromium™ Single Cell 3′ Library & Gel Bead Kit v2, PN-120237, a Single Cell 3′ Chip Kit v2 PN-120236, and an i7 Multiplex Kit PN-120262” (10× Genomics)^60^, following the user guide from the Single Cell 3′ Reagent Kit v2 (Manual Part # CG00052 Rev A). Libraries were run on an Illumina HiSeq 4000 system as 150 bp paired-end reads, one full lane per sample.

### Single-cell RNAseq data pre-processing

Sequencing results were demultiplexed and converted to FASTQ format using Illumina bcl2fastq software. The Cell Ranger Single-Cell Software Suite (https://support.10xgenomics.com/single-cell-gene expression /software/ pipelines/latest/ what-is-cell-ranger) was used to perform sample demultiplexing, barcode processing, and single-cell 3′ gene counting. The cDNA insert was aligned to the mm10/GRCm38 reference genome. Only confidently mapped, nonPCR duplicates with valid barcodes and UMIs were used to generate the gene-barcode matrix containing 10,654 cells (4,768 Siah WT and 5,886 *Siah2*^*−/−*^). Further analysis, including quality filtering, identification of highly variable genes, dimensionality reduction, standard unsupervised clustering algorithms, and discovery of DEGs, was performed using the Seurat R package^61^. To exclude low quality cells as well as cells that were extreme outliers in terms of library complexity or that may include multiple cells or doublets, we calculated the distribution of the number of detected genes per cell and removed cells in the bottom and top 2% quantiles. We also removed cells with >10% of transcripts derived from mitochondrial genes. After quality filtering, the mean and median number of detected genes per cell was 2847.6 and 2575, respectively. After removing unwanted cells from the dataset, we normalized the data by the total expression, multiplied by a scale factor of 10,000, and log-transformed the result.

### Integrated analysis of single-cell datasets

To account for batch differences, we utilized the Seurat alignment method for data integration^62^, which does not expect that confounding variables have uniform effects on all cells in a dataset and allows for global transcriptional shifts between datasets. The method uses a variant of canonical correlation analysis (CCA) to find linear combinations of features and identifies shared correlation structures across datasets^62–64^. For each dataset, we identified variable genes, while controlling for a strong relationship between variability and average expression. We took the union of the top 2000 genes with the highest dispersion from both datasets and ran CCA to determine common sources of variation between the two datasets. We then aligned the subspaces based on the first 20 canonical correlation vectors, generating a new dimensional reduction used for further analysis. Original single-cell data has been deposited in the public dataset, GSE 134814.

### Visualization and clustering

To visualize data, we further reduced the dimensionality of the entire 10075 cell dataset to project cells in two-dimensional space using *t*-SNE based on the aligned CCA. Aligned CCA was also used as a basis for partitioning the dataset into clusters using a shared nearest neighbor modularity optimization algorithm. Using graph-based clustering, we divided cells into 19 transcriptionally similar subpopulations. We merged biologically similar clusters, resulting in 16 defined subpopulations. We identified nonimmune populations based on expression of Ptprc.

### Determining cluster markers

To identify cluster markers, we performed pairwise differential expression analysis using the Model-based Analysis of Single-cell Transcriptomics (MAST) method^65^, adjusting for cellular detection rate, for each cluster against all other clusters for autosomal genes detected in at least 20% of cluster cells, keeping significant genes in each comparison. Significance was determined using the MAST method with Bonferroni multiple-comparison correction. Immune cell types within clusters were identified based on expression of known markers. Additionally, pathway analysis was performed for significantly expressed genes in each cluster using Enrichr (http://amp.pharm.mssm.edu/Enrichr/) to confirm cell types.

### Cell cycle classification

We calculated a module score for each cell based on average expression levels of G2/M and S phase markers, subtracted by the aggregated expression of randomly selected control genes, as implemented in the Seurat R package^66^. Each cell was assigned to the cell cycle phase with the highest positive score, and to G1 for negative scores. The same scoring approach was also used to quantify pathway enrichment.

### T cell stimulation assay

Lymphocytes from draining lymph nodes or T cells isolated from spleen were cultured in T-cell medium (DMEM supplemented with 10% FBS, penicillin/streptomycin, 1% HEPES, 1% nonessential amino acids, 100 mM sodium pyruvate, mercaptoethanol (1000×; SIGMA) plus 1 μg/ml of CD28 (BioLegend) and 100 U/ml rhIL-2 (Fisher Scientific) in CD3^−^-coated (5 μg/ml) plates for 72 h at 37° C, at a cell density of 10^6^/ml.

### T cell isolation

Spleens were minced and passed through a 70-μm cell strainer to generate a single-cell suspension. Red blood cells were removed by incubation in RBC lysing buffer (Sigma). To purify CD4^+^ and CD8^+^ T cells, splenocytes were incubated with biotinylated anti-B220 (RA3^−^6B2, 1:300), biotinylated anti-CD11b (M1/70, 1:300), biotinylated anti-CD11c (N418, 1:300), biotinylated anti-CD19 (MB19-1, 1:300), biotinylated anti-CD24 (M1/69, 1:500), and anti-CD16/32 (93, 1:75), all from BioLegend for 20 min at room temperature, followed by incubation with appropriate reagents from the EasySep mouse streptavidin RapidSpheres isolation kit (Stem Cell Technologies), according to manufacturer’s protocol. Collected supernatants were enriched with T cells, and the purity of CD4^+^ and CD8^+^ cells was 92–97% based on FACS analysis.

### Mouse Treg transduction

A total of 500,000 Treg cells in 96-well plate were grown in RPMI supplemented with 10% FBS, 1% HEPES, 0.05% 2-mercaptoethanol, and Amp/Pen in presence of IL-2 (100 U/ml) and anti-CD3 anti-CD28-coated microbeads with bead to cell ratio 1:1. shRNAs were purchased from Sigma-Aldrich mouse p27#12 TRCN0000071063; mouse p27#2 TRCN0000071067; non targeted shRNA control; SHC016. Freshly collected supernatants containing lentiviral particles were added and supplemented with polybrene. Infection was performed by centrifugation. Fresh media containing IL-2 and anti-CD3 anti-CD28-coated microbeads was added after 24 h, along with the selective agent puromycin (Sigma). After 48 h, the media was replaced with fresh media containing IL2 and anti-CD3 anti-CD28-coated microbeads, and cells were collected for mRNA analysis 1 week after infection.

### Mouse Treg in vitro expansion

Isolated Tregs (CD4^+^ CD25^*+*^ cells) were expanded using Dynabeads mouse T activator CD3/CD28 (Gibco Life Technologies) kit and related protocol.

### Treg isolation

Spleens were minced and passed through a 70-μm cell strainer to generate a single-cell suspension. CD4-positive cells were purified using mouse CD4^+^ T cell isolation kit (Easy Sep Mouse CD4^+^ T cell isolation kit Cat# 19852, Stemcell), followed by sorting of Treg cells using CD4 and CD25 as markers.

### DC isolation

Tumors were excised, minced, and digested with 1 mg/ml collagenase D (Roche) and 100 µg/ml DNase I (Sigma) at 37 °C for 1 h. Digests were then passed through a 70-μm cell strainer to generate a single-cell suspension. Myeloid cells were isolated by centrifugation over the density gradient media Lymphoprep (Axis-Shield), followed by sorting of DCs using the marker CD11c.

### Treg suppression assay

CD4^+^ CD25^*−*^ T cells and CD4^+^ CD25^+^ T cells were purified from WT C57BL/6 mice using mouse a CD4^+^ T cell isolation kit (Easy Sep Mouse CD4^+^ T cell isolation kit Cat# 19852, Stemcell) and an Easy Sep Mouse CD25 Regulatory T cell positive selection kit (Cat#18782, Stemcell), and subsequently labeled with CFSE (Cell Trace CFSE Cell Proliferation Kit Cat# C34554 Molecular Probes, Inc). Briefly, purified T cells were suspended in PBS (+0.1% BSA) at 10^7^/ml with 5 µM CFSE, incubated for 10 min at 37 °C and then washed in complete T cell medium. Purified T cells were cultured at 2 × 10^5^/well in 96 well plates precoated with 2 µg/ml anti-CD3 (eBioscience; 16-0031-82) and 2 µg/ml anti-CD28 (eBioscience; 16-0281-85), along the indicated ratio (2:1, 4:1, 8:1, 16:1, (Tconv:Treg)) of unlabeled WT or *Siah2*^*−/−*^ CD4^+^CD25^+^ Tregs in complete T cell medium. After 3 days of incubation, stained cells were analyzed with LSR Fortessa X20 (Becton Dickenson).

### Analysis of human melanoma tumors

Correlation of *SIAH2* expression and that of Treg signature genes identified in murine models (*FOXP3, CD8A, GZMA, GZMB, IFNG, CCL4, CCL3, CCL2, CCL5, IL1B, CXCL9, NOS2, CCL17,* and *CCL22*) was evaluated using Spearman’s rank correlations on high (*N* = 66) and low (*N* = 139) immune subgroups of metastatic melanoma samples (*N* = 339; TCGA_SKCM metastatic samples^48^). Rho and *P* values were used. The cutoff for significant/suggestive correlations was *R* ≥ |0.2|.

### Statistical Analysis

GraphPad Prism version 7 was used for statistical analysis. Differences between two groups were assessed using two-tailed unpaired *t*-test or Wilcoxon rank-sum test. Two-way analysis of variance (ANOVA) with Bonferroni’s multiple comparison test was used to evaluate experiments involving multiple groups. *P < 0.05; **P < 0.01; ***P < 0.001; and ****P < 0.0001. For analysis of human metastatic melanoma samples Rho and *P* values were used and the cutoff for significant/suggestive correlations was *R* ≥ |0.2|.

### Reporting summary

Further information on research design is available in the Nature Research Reporting Summary linked to this article.

## Supplementary information

Supplementary Information

Reporting Summary

## Data Availability

RNAseq data has been deposited in Gene Expression Omnibus (GEO) under accession number GSE134412. NanoString data is available in GEO under accession number GSE134328. Single-cell RNAseq data is available in GEO under accession number GSE134814. The source data underlying Figs. 1a, c, d, 2, 3, 4c–g, 5 and 6a–i, and Supplementary Figs. 1b, c, 2, 3, 5a, b, d–l and 6a–f are provided in the source data file. Raw data for westerns blots is provided in the Supplementary Information. The results shown here are in part based upon data generated by the TCGA Research Network as part of the TCGA_SKCM data set.

## Source Data

Source Data
