# Supplementary material for: Siah2 control of T-regulatory cells limits anti-tumor immunity
Source: Nat Commun. 2020 Jan 7;11:99. doi: 10.1038/s41467-019-13826-7 (PMC6946684; doi:10.1038/s41467-019-13826-7)
Supplement: Supplementary file 1 — Supplementary Information [file 41467_2019_13826_MOESM1_ESM.pdf]

Siah2 control of T-regulatory cells limits anti-tumor immunity

Supplemental Figures (6)

Supplemental Tables (5)

Original Western Blots (3)

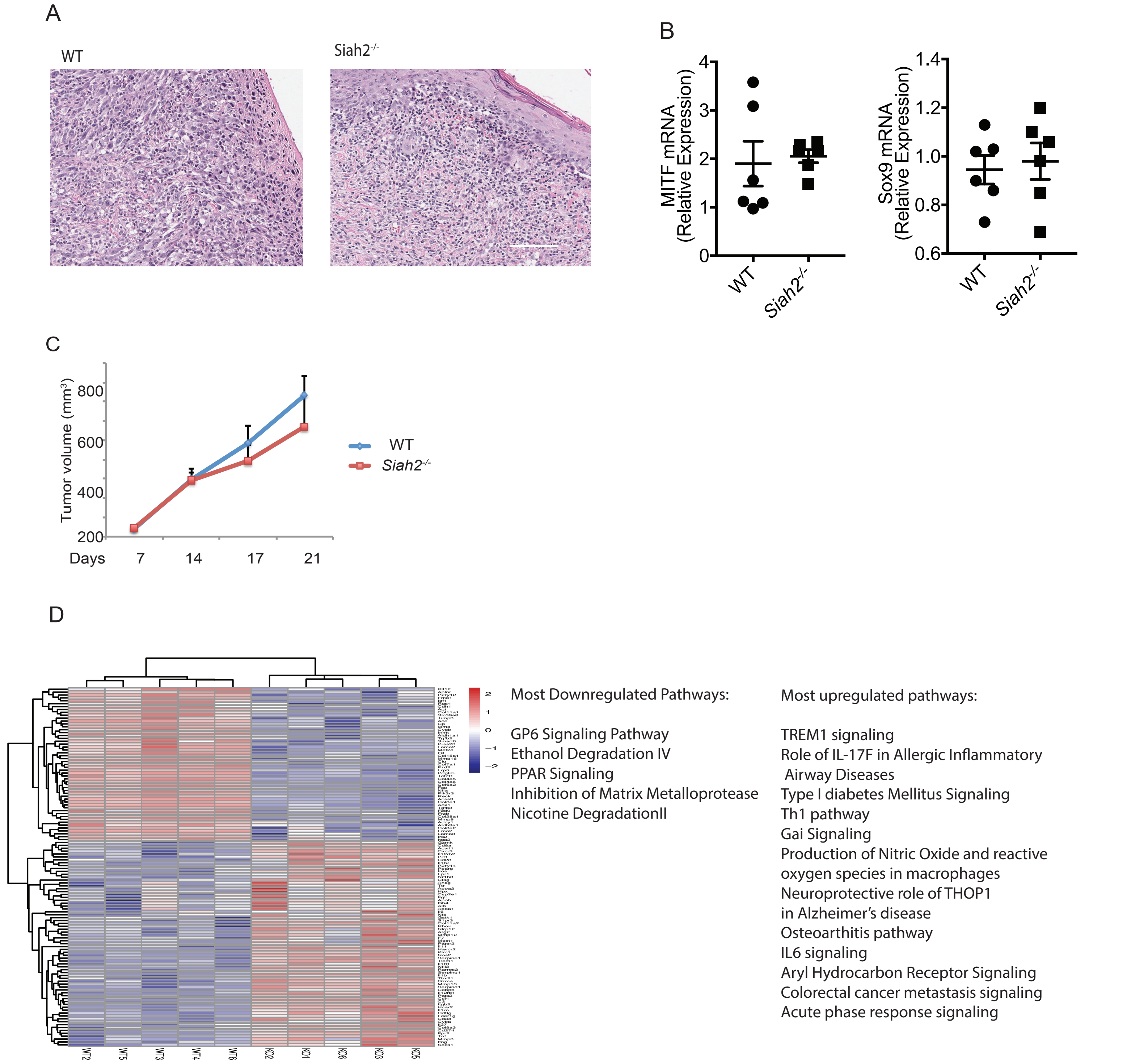

Supplemental Figure 1. Melanomas grown in $Siah2^{-/-}$ mice exhibit immune phenotype.
(**A**) YUMMER1.7 cells ($4x10^5$) were injected into the flank of 5-7 weeks old Siah2 WT or *$Siah2^{-/-}$* male mice. H&E staining was performed on tumors collected after 11 days, Scale bar, 100 μm. (**B**) qPCR analysis of indicated mRNAs was performed on tumors collected 11 days after injection (n=6, both genotypes, Mean ± s.e). (**C**) YUMMER1.7 cells (10^6) were injected into the flank of 5-7 weeks-old Siah2 WT or *$Siah2^{-/-}$* male mice and tumor growth (volume) was assessed over time. N=4 for both genotypes, Mean ± s.e. (**D**) RNAseq analysis of tumors from both genotypes collected 11 days after melanoma cell injection. Heat map shows all the genes related to the significant up/downregulated pathways using the pathway criteria Z-score> 1 and p value < 0.01 (n=5).

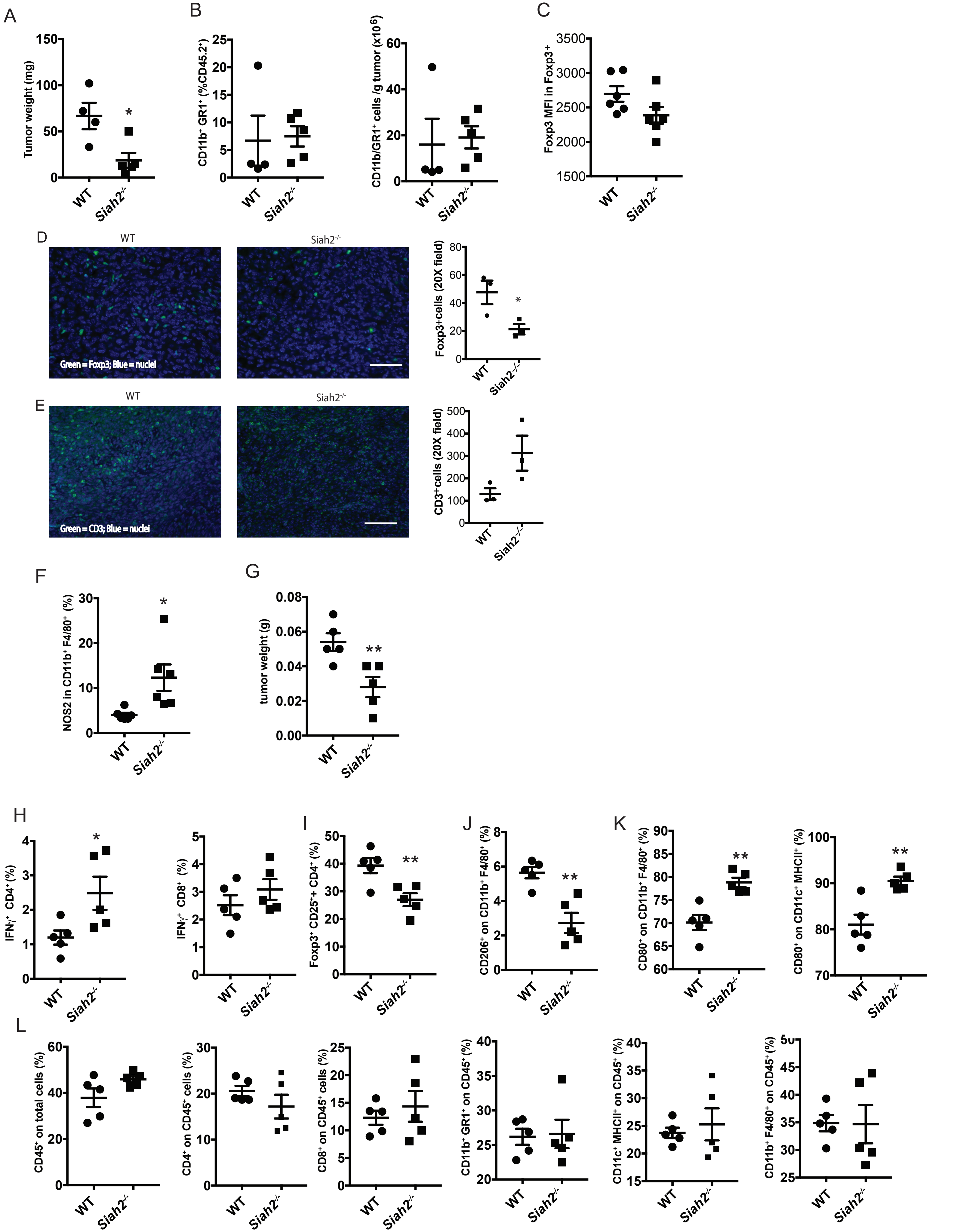

Scortegagna et al. Sup Figure 2

Supplemental Figure 2.

Siah2 loss in tumor microenvironment increases frequency of intra-tumoral effector T cells and decreases tumor-infiltrating Tregs.
(**A**) Weights of tumors from YUMMER1.7 melanoma cell-injected in Siah2 WT or Siah2-/- mice collected 11 days after injection (WT n=4; KO n=5). (**B**) Quantification of CD11B^{+} GR1^{+} cells on day 11 after tumor injection represented as total number/g tumor tissue or percentage of CD45.2^{+} immune cells (WT n=4; KO n=5). (**C**)FOXP3 expression in CD4^{+} FOXP3^{+} cells based on flow cytometry (n=6) on day 11 after tumor cells injection. FOXP3 (**D**) and CD3 (**E**) staining of tumors from Siah2 WT or *Siah2*$^{-/-}$ mice, as assessed 11 days after melanoma cells injection (left panels), plus quantification (right) (n=3). Scale bar, 100 µm. (**F**) Percentage of NOS2^{+} cells among CD11b/F480^{+} cells on day 11 after tumor cells injection (WT n=4; KO n=5). (**G**) Weights of tumors from YUMM1.7 cell-injected in Siah2 WT or *Siah2*$^{-/-}$ mice, collected 11 days after injection (n=5). (**H**) Frequencies of tumor-infiltrating IFN γ-producing CD8^{+} and CD4^{+} T cells on day 11 after tumor inoculation and following overnight stimulation in vitro with melanoma peptides (n=5) . Frequencies of tumor-infiltrating Foxp3^{+} CD25^{+} cells within the CD4^{+} T cell population (**I**), CD206^{+} cells among CD11b/F4/80-positive cells (**J**), and CD80^{+} cells among CD11b/F4/80^{+} and CD11c^{+} MHCII^{+} cells (**K**) on day 11 after tumor inoculation (n=5). (**L**) Frequencies of tumor-infiltrating CD45^{+} among total cells or CD4^{+}, CD8^{+}, CD11B^{+} GR1^{+} and CD11b^{+}/F4/80^{+}, CD11c^{+} MHCII^{+} cells among CD45^{+} on day 11 after tumor injection (n=5). Mean ± s.e. Data were analyzed by unpaired t-test. **$P < 0.005$, *$P < 0.05$ compared with WT.

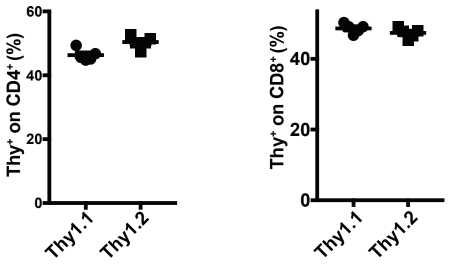

Supplemental Figure 3. Frequencies of Thy1.1, Thy1.2 markers in CD4⁺ and CD8⁺ cells in blood of transplanted mice.
Frequencies of cells expressing Thy1.1 and Thy1.2 markers among $CD4^+$ and $CD8^+$ cells from blood samples of mice transplanted 8 weeks earlier with mixed BM from WT (Thy1.1) and $Siah2^{-/-}$ (Thy1.2) mice, prior to tumor cell injection (n=5), Mean ± s.e.

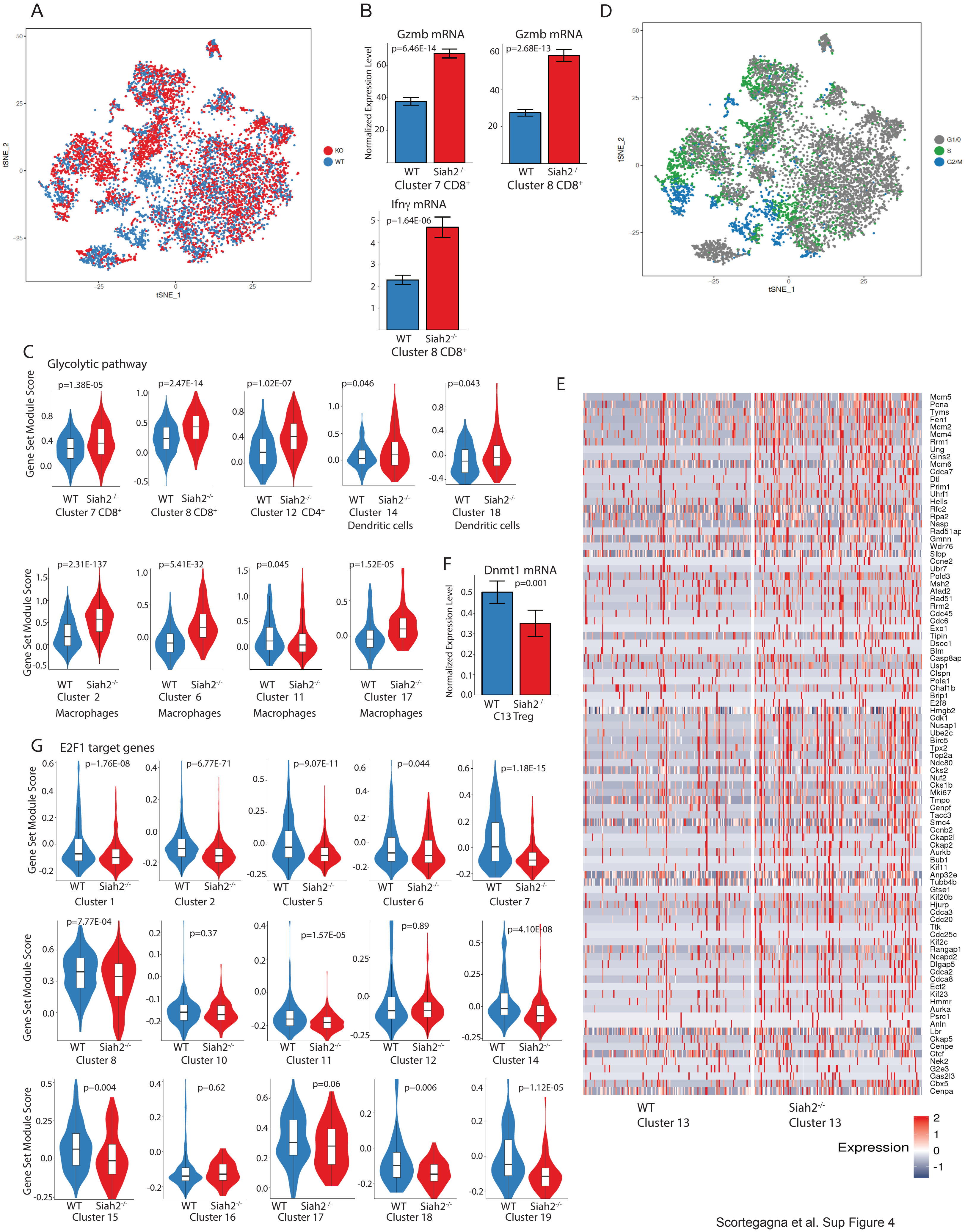
A
tSNE_1
tSNE_2
KO
WT
B
Gzmb mRNA
p=6.46E-14
p=2.68E-13
Normalized Expression Level
WT Siah2-/-
Cluster 7 CD8+
Cluster 8 CD8+
Ifnγ mRNA
p=1.64E-06
D
G1/0
S
G2/M
C
Glycolytic pathway
Gene Set Module Score
p=1.38E-05
p=2.47E-14
p=1.02E-07
p=0.046
p=0.043
Cluster 12 CD4+
Cluster 14 Dendritic cells
Cluster 18 Dendritic cells
p=2.31E-137
p=5.41E-32
p=0.045
p=1.52E-05
Cluster 2 Macrophages
Cluster 6 Macrophages
Cluster 11 Macrophages
Cluster 17 Macrophages
E
F
Dnmt1 mRNA
p=0.001
C13 Treg
G
E2F1 target genes
p=1.76E-08
p=6.77E-71
p=9.07E-11
p=0.044
p=1.18E-15
p=7.77E-04
p=0.37
p=1.57E-05
p=0.89
p=4.10E-08
p=0.004
p=0.62
p=0.06
p=0.006
p=1.12E-05
Cluster 1
Cluster 2
Cluster 5
Cluster 6
Cluster 7
Cluster 8
Cluster 10
Cluster 11
Cluster 12
Cluster 14
Cluster 15
Cluster 16
Cluster 17
Cluster 18
Cluster 19
Mcm5 Pcna Tyms Fen1 Mcm2 Mcm4 Rrm1 Ung Gins2 Mcm6 Cdca7 Dtl Prim1 Uhrf1 Hells Rfc2 Rpa2 Nasp Rad51ap Gmnn Wdr76 Slbp Ccne2 Ubr7 Pold3 Msh2 Atad2 Rad51 Rrm2 Cdc45 Cdc6 Exo1 Tipin Dscc1 Blm Casp8ap Usp1 Clspn Pola1 Chaf1b Brip1 E2f8 Hmgb2 Cdk1 Nusap1 Ube2c Birc5 Tpx2 Top2a Ndc80 Cks2 Nuf2 Cks1b Mki67 Tmpo Cenpf Tacc3 Smc4 Ccnb2 Ckap2l Ckap2 Aurkb Bub1 Kif11 Anp32e Tubb4b Gtse1 Kif20b Hjurp Cdca3 Cdc20 Ttk Cdc25c Kif2c Rangap1 Ncapd2 Dlgap5 Cdca2 Cdca8 Ect2 Kif23 Hmmr Aurka Psrc1 Anln Lbr Ckap5 Cenpe Ctcf Nek2 G2e3 Gas2l3 Cbx5 Cenpa
WT Cluster 13
Siah2-/- Cluster 13
Expression
Scortegagna et al. Sup Figure 4

Supplemental Figure 4. Increased E2F1 and glycolytic pathway genes in *Siah2*$^{-/-}$ immune cells clusters.
(**A**) tSNE plot of CD45^{+} cells from YUMMER1.7 melanoma tumors collected 12 days after their inoculation into WT or *Siah2*$^{-/-}$ mice showing WT and *Siah2*$^{-/-}$ distribution within each cluster. (**B**) mRNA expression of indicated genes identified by single-cell RNAseq within the indicated clusters, both genotypes . (**C**) Violin plot for expression levels of genes involved in the glycolytic pathway determined by single-cell RNAseq in different clusters. (**D**) tSNE plot of CD45^{+} cells from YUMMER1.7 melanoma tumors collected 12 days after their inoculation into WT or *Siah2*$^{-/-}$ mice showing distribution of the different cell cycle phases within each cluster . (**E**) Heat map showing scaled expression values of genes involved in cell cycle. (**F**) mRNA expression of DNMT1 identified by single-cell RNAseq within the Treg cluster (C13), both genotypes. (**G**) Violin plot for expression levels of E2F1 target genes determined by single-cell RNAseq in different clusters. P value is from Wilcoxon rank-sum test. Mean $\pm$ s.e.

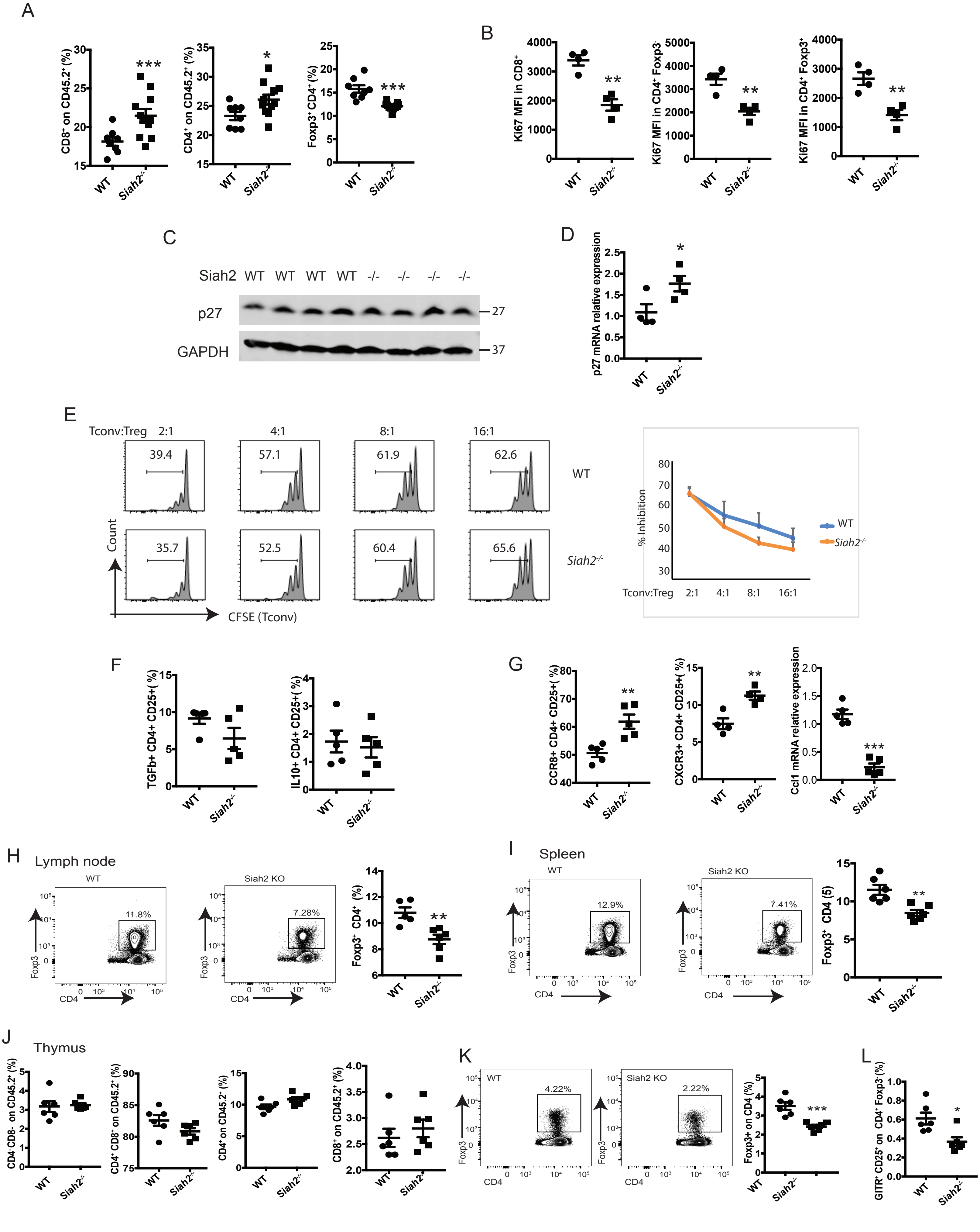

Scortegagna et al. Sup Figure 5

Supplementary Figure 5.

Comparable Treg frequency in tumor free WT and *Siah2$^{-/-}$* mice; comparable treg suppression function in vitro in *Siah2$^{-/-}$* and WT treg.
(**A**) Draining lymph nodes from Siah2 WT and *Siah2$^{-/-}$* mice were collected and the percentage of $CD4^+$ and $CD8^+$ cells among $CD45.2^+$ cells, plus the percentage of $Foxp3^+$ cells in $CD4^+$ cells were determined by flow cytometry (WT n=8; *Siah2$^{-/-}$* n=11). (**B**)Lymphocytes were cultured and stimulated with CD3/CD28 antibodies in presence of IL2, and 3 days later assessed for Ki67 expression in $CD8^+$, $CD4^+$ $Foxp3^-$, $CD4^+$ $Foxp3^+$ cells based on flow cytometry (n=4). (**C**) Lysates made from unstimulated lymphocytes derived from draining Lymph nodes were prepared and immunoblotted for p27 and GAPDH (n=4). (**D**) q-RTPCR analysis of p27 mRNA from lymphocytes stimulated as in panel B. n=4, both genotypes. (**E**) In vitro suppression assay of conventional $CD4^+$ T cells (Tconv.) labeled with cytosolic dye carboxyfluorescein diacetate succinimidyl ester (CFSE) in the presence of WT or *Siah2$^{-/-}$*Treg cells ($CD4^+$ $CD25^+$) and quantification (right panel). Numbers indicate the percentage of cells in the indicated area. Conventional $CD4^+$ T cell division was analyzed by CFSE dilution at the indicated ratios of Tconv and Treg cells following 3 day stimulation with plate bound anti-CD3 andanti-CD28 (both 2ug/mL). Graph on the right panel is the average percent inhibition (100 - % divided) of three individual experiments (five biological replicates) of WT versus *Siah2$^{-/-}$* Treg cells. (**F**) Frequencies of tumor-infiltrating $TGF\beta^+$ or $IL10^+$ $CD4^+/CD25^+$ T cells 11 days after tumor cells inoculation and following overnight stimulation in vitro with PMA and Ionomicin (n=5). (**G**) Left two panels:Percentage of $CCR8^+$ and $CXCR3^+$ cells among $CD4/CD25^+$ cells on day 11 after tumor cells injection (n=5 for $CCR8^+$ and n=4 for $CXCR3^+$ cells), right panel: qPCR analysis of Ccl1 mRNA was performed on tumors collected 11 days after injection (n=5, both genotypes, Mean ± s.e). Frequencies of $Foxp3^+$ cells within the CD4 population from the lymph nodes (**H**) and spleen (**I**) of tumor-free mice (n=5). (**J**) Frequencies of CD4/CD8 double negative, CD4/CD8 double-positive, and single-positive CD4 or CD8 cells from thymus of Siah2 WT and *Siah2$^{-/-}$* mice (n=5). Frequencies of Foxp3-positive cells in the CD4 population (**K**) and $GITR^+$ $CD25^+$ in the $CD4^+$ $Foxp3^-$ population (**L**) from thymus of Siah2 WT and *Siah2$^{-/-}$* mice (n=5). Mean ± s.e. Data were analyzed by unpaired t-test. ***$P < 0.0005$, **$P < 0.005$, *$P < 0.05$ compared with WT.

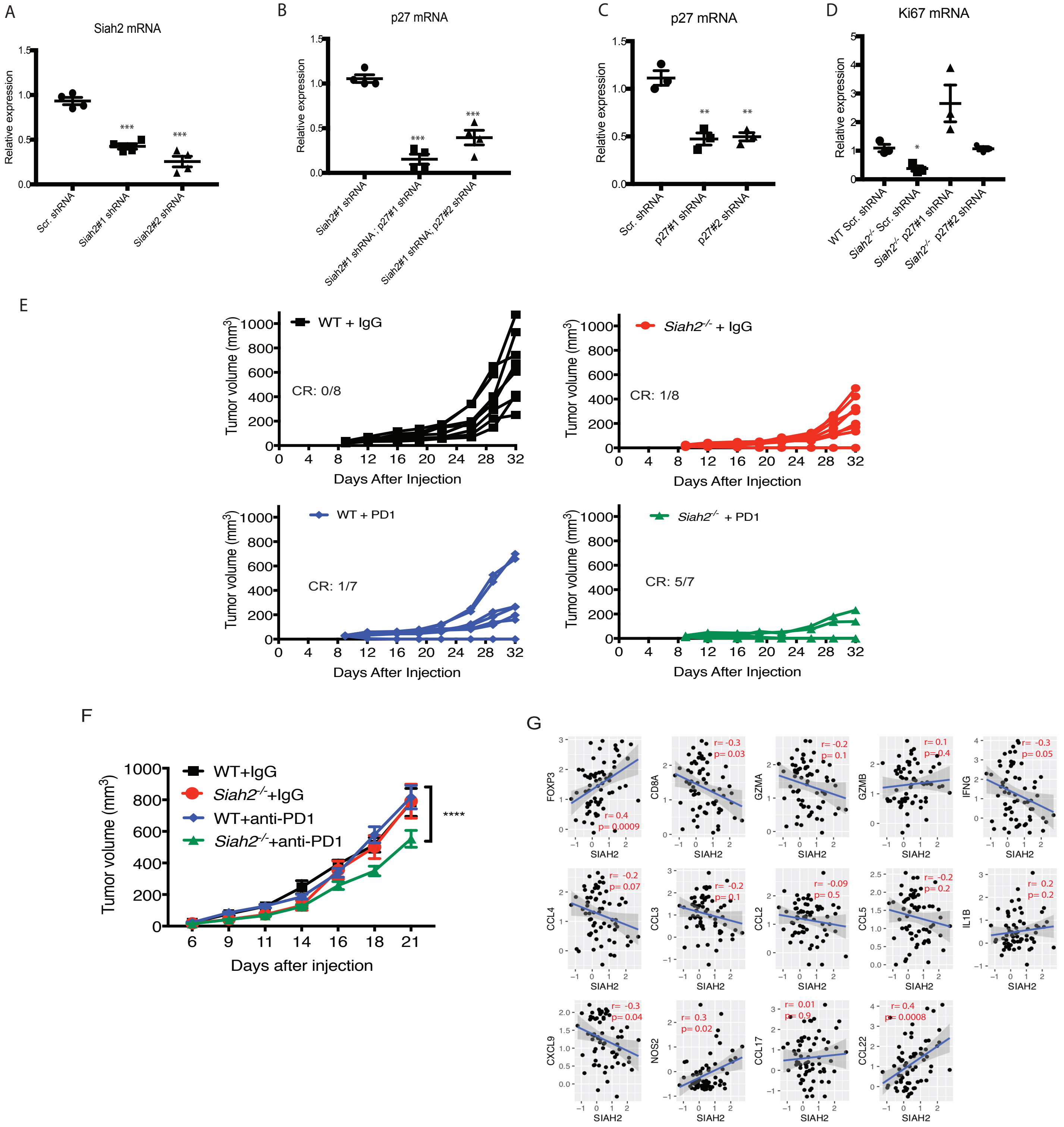

Supplemental Figure 6. Effective PD1 therapy in Siah2$^{-/-}$ grown tumors; Siah2 expression in immune responsive melanomas. **A** and **B**, growth inhibition upon Siah2 deletion is p27 dependent. q-RTPCR analysis of Siah2 (A) and p27 (B) mRNA from Jurkat cells depleted of Siah2 and p27 after infection with shRNA encoding lentiviruses. Mean ± s.e., n=4. **C** and **D**, growth inhibition upon Siah2 deletion in Treg cells is p27 dependent: q-RTPCR analysis of p27 (C) and Ki67 (D) mRNA from isolated WT or *Siah2$^{-/-}$* Treg cells in which p27 was knocked down after infection with shRNA encoding lentiviruses. Mean ± s.e., n=3. **E**, individual (J) tumor growth curves over time of YUMM1.7 cells (150,000) inoculated in WT and Siah2$^{-/-}$ mice and treated with anti-PD1 antibody. Mice were treated with either rat isotype (IgG) or anti-PD1 antibody (200 µg/ mouse; 3 times per week for a total of 5 times) starting from day 7 after melanoma cells injection. WT and Siah2$^{-/-}$ IgG (n=8); WT and Siah2-/- anti-PD1 (n=7). Complete regression (CR) rates at study termination are shown (J). **F,** MC38 cells (500,000) were inoculated in WT (n=8) and Siah2$^{-/-}$ (n=7) mice. Seven days later, mice were subjected to administration of anti-PD1 antibody(100 µg/ mouse) or rat isotype (IgG) 3 times per week, for total of 4 injections. Mean ± s.e. data were analyzed by unpaired t-test (A-D) or two-way ANOVA with Bonferroni multiple comparison (F). ***P < 0.0005, **P < 0.005, *P < 0.05 compared to Scr control. shRNA (A, C, D), Siah2#1 shRNA (B), WT + anti-PD1 (F). **G,** Correlation of Siah2 and Foxp3 expression in high immunogenic human melanoma specimens; Shown are Spearman's rank correlations that are indicated for each gene (R and P values).

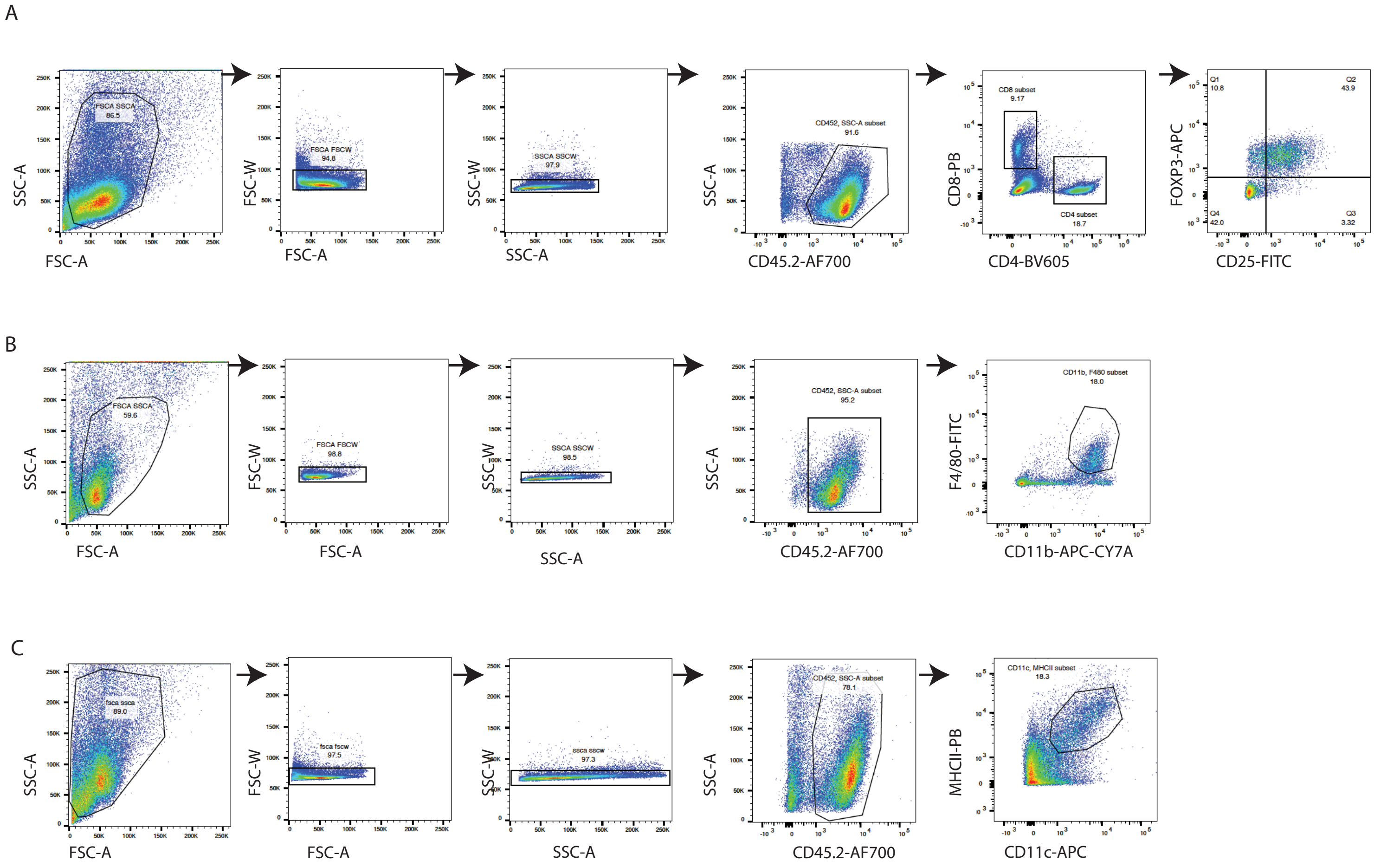

Supplementary Figure 7. Gating strategy for immune cells analysis. (**A**) Gating strategy of the tumor infiltrating CD45$^+$, CD4$^+$, CD8$^+$, FOXP3$^+$ CD25$^+$ cells. (**B**) Gating strategy of the tumor infiltrating CD11b$^+$F4/80$^+$ cells. (**C**) Gating strategy of the tumor infiltrating CD11c$^+$ MHCII$^+$ cells.

Supplemental Table 1: List of primers used for QPCR analysis

**Species**	**Gene**	**Primer Forward**	**Primer Reverse**
mouse	Ccl17	TACCATGAGGTCACTTCAGATGC	GCACTCTCGGCCTACATTGG
mouse	Nos2	GTTCTCAGCCCAACAATACAAGA	GTGGACGGGTCGATGTCAC
mouse	IFNg	GCCACGGCACAGTCATTGA	TGCTGATGGCCTGATTGTCTT
mouse	Cxcl9	GGAGTTCGAGGAACCCTAGTG	GGGATTTGTAGTGGATCGTGC
mouse	Ccl22	AGGTCCCTATGGTGCCAATGT	CGGCAGGATTTTGAGGTCCA
mouse	Foxp3	CACCTATGCCACCCTTATCCG	CATGCGAGTAAACCAATGGTAGA
mouse	Cdkn1b (p27)	TCAAACGTGAGAGTGTCTAACG	CCGGGCCGAAGAGATTTCTG
mouse	Ccne1 (Cyclin E1)	CTCCGACCTTTCAGTCCGC	CACAGTCTTGTCAATCTTGGCA
mouse	TK1	AGTGCCTGGTCATCAAGTATGC	GCTGCCACAATTACTGTCTTGC
mouse	Mki67	ATCATTGACCGCTCCTTTAGGT	GCTCGCCTTGATGGTTCCT
mouse	Dnmt1	ACTCCCTTCGGGCATAGCAT	AGGTTGCAGACGACAGAACAG
mouse	Cdkn1b (p27)	TCAAACGTGAGAGTGTCTAACG	CCGGGCCGAAGAGATTTCTG
human	siah2	TCTTCGAGTGTCCGGTCTG	CGGCATTGGTTACACACCAG
human	Cdkn1b (p27)	AACGTGCGAGTGTCTAACGG	CCCTCTAGGGGTTTGTGATTCT
mouse	Ccl1	GGCTGCCGTGTGGATACAG	AGGTGATTTTGAACCCACGTTT

Supplemental Table 2: List of genes used in glycolytic pathway

**Genes in glycolytic pathway**
Hk1
Hk2
Hk3
Gpi1
Pfkl
Pfkp
Aldoa
Tpi1
Gapdh
Pgk1
Pgam1
Eno1
Pkm2
Ldha

Supplemental Table 3: List of genes upregulated by E2F1

List of genes upregulated by E2F1
Ccne1
Tk1
Mki67
Dnmt1
Cdk1
Ccna1
Cdc6
Ccnd1
Ccnd3
Cdc25a
Cdk2
E2f1
Cdc7
Mcm2
Mcm3
Mcm4
Mcm5
Mcm6
Orc1
Rfc2
Rfc3
Rfc4
Rrm1
Rrm2
Top2a
Tyms

Supplemental Table 4: List of genes associated with IFNγ/IL12 pathway

**genes associated with IFNγ/IL12 pathway**
Cd28
Cd3d
Cd3e
Cd86
Prf1
Stat3
Jun
H2-Eb1
Il2ra
Etv5
Fos
IL2
Cd80
Irf1
Crebbp
Ppp3ca
Mapk8
Pias2
Cd247
Cd4
Ppp3cb
Cd3g
Ppp3r1
Il18r1
Stat4
Mapk9
Tgfb1
Tbx21
Il18rap
Ifng
Il18

Supplemental Table 5: Number and frequency of cells across *CD45+ (Ptprc-expressing) clusters*, separated by sample (Siah2-/- or WT).

cluster	sample	num_cd45+_sample	num_cells_cluster_sample	pct_cd45+_cluster_sample
C01	KO	5349	687	12.84
C01	WT	3741	406	10.85
C02	KO	5349	1966	36.75
C02	WT	3741	1424	38.06
C05	KO	5349	552	10.32
C05	WT	3741	203	5.43
C06	KO	5349	244	4.56
C06	WT	3741	417	11.15
C07	KO	5349	414	7.74
C07	WT	3741	214	5.72
C08	KO	5349	318	5.95
C08	WT	3741	285	7.62
C11	KO	5349	307	5.74
C11	WT	3741	138	3.69
C12	KO	5349	324	6.06
C12	WT	3741	52	1.39
C13	KO	5349	150	2.80
C13	WT	3741	182	4.87
C14	KO	5349	161	3.01
C14	WT	3741	169	4.52
C17	KO	5349	60	1.12
C17	WT	3741	134	3.58
C18	KO	5349	85	1.59
C18	WT	3741	58	1.55
C19	KO	5349	81	1.51
C19	WT	3741	59	1.58

Original Full Western Blots

Figure 5C

Figure 6A

Figure 6C

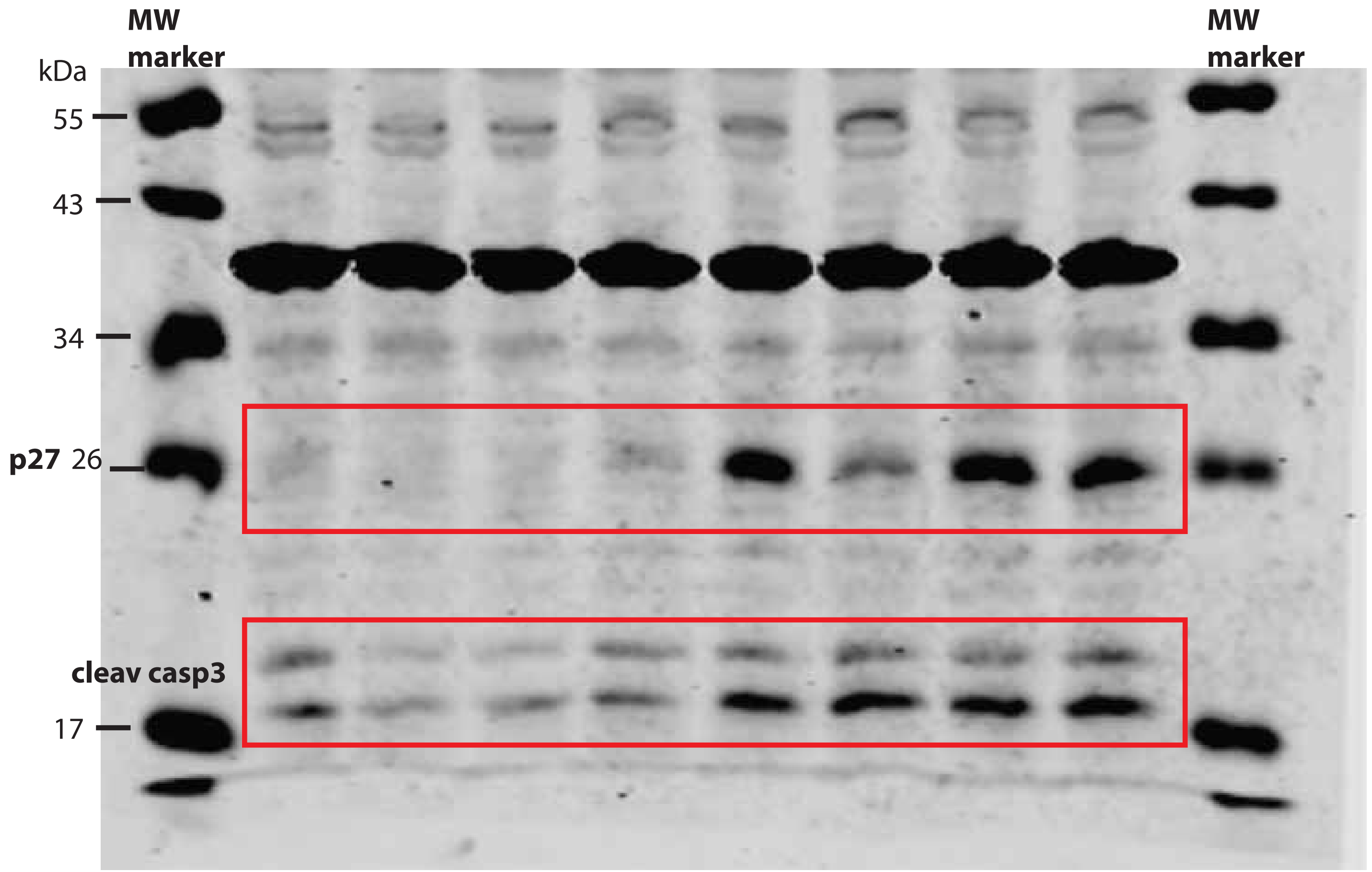

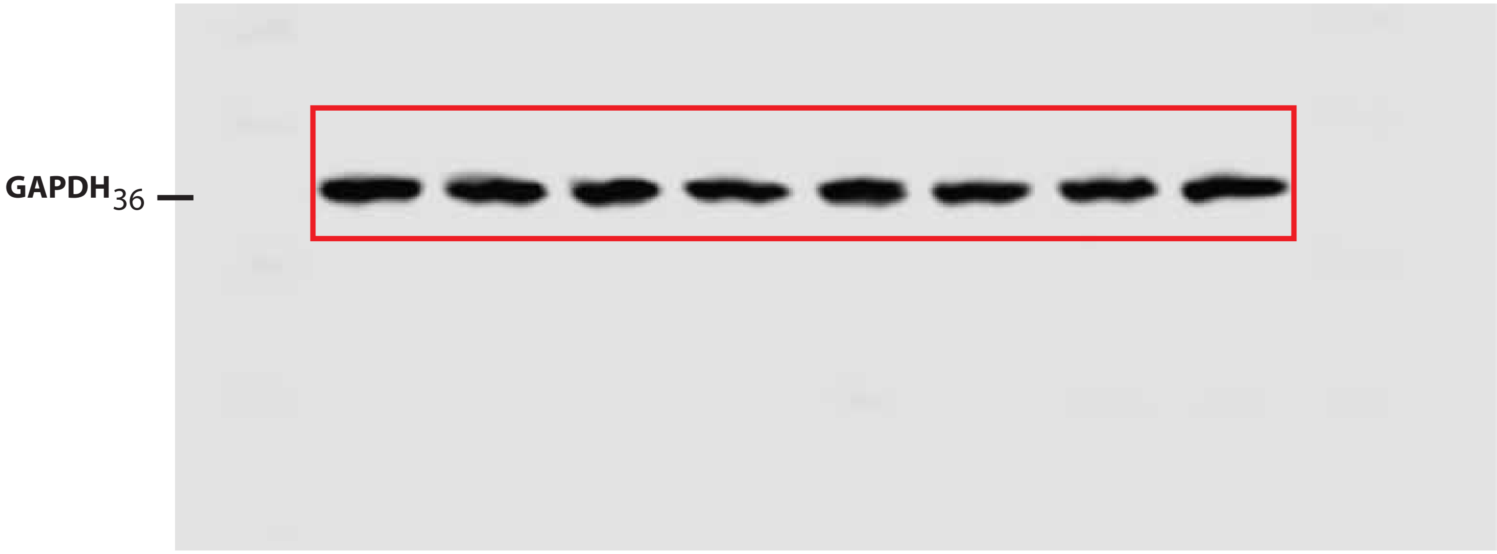

Western blot data for figure 5C

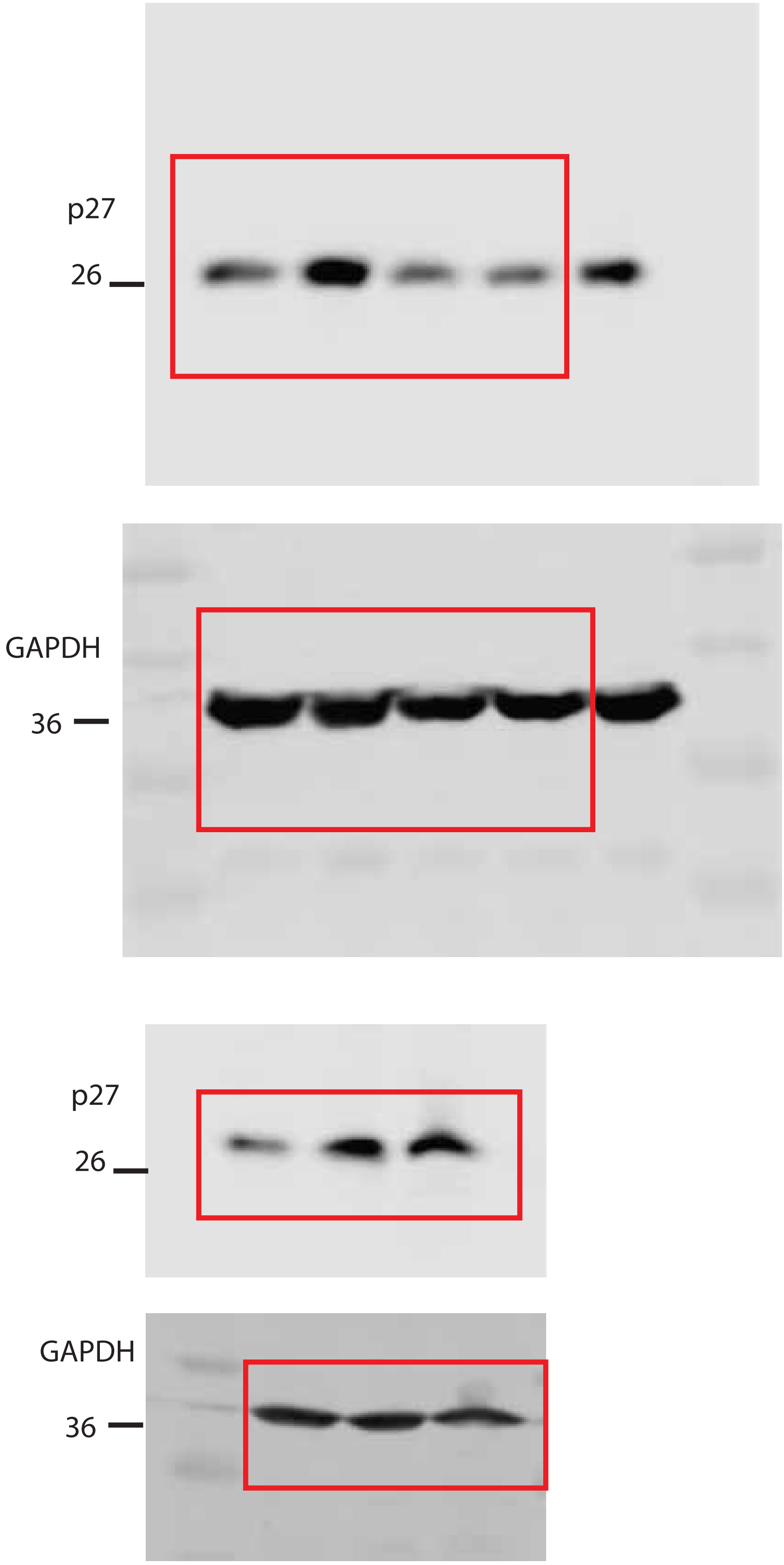

Western blot data for figure 6A

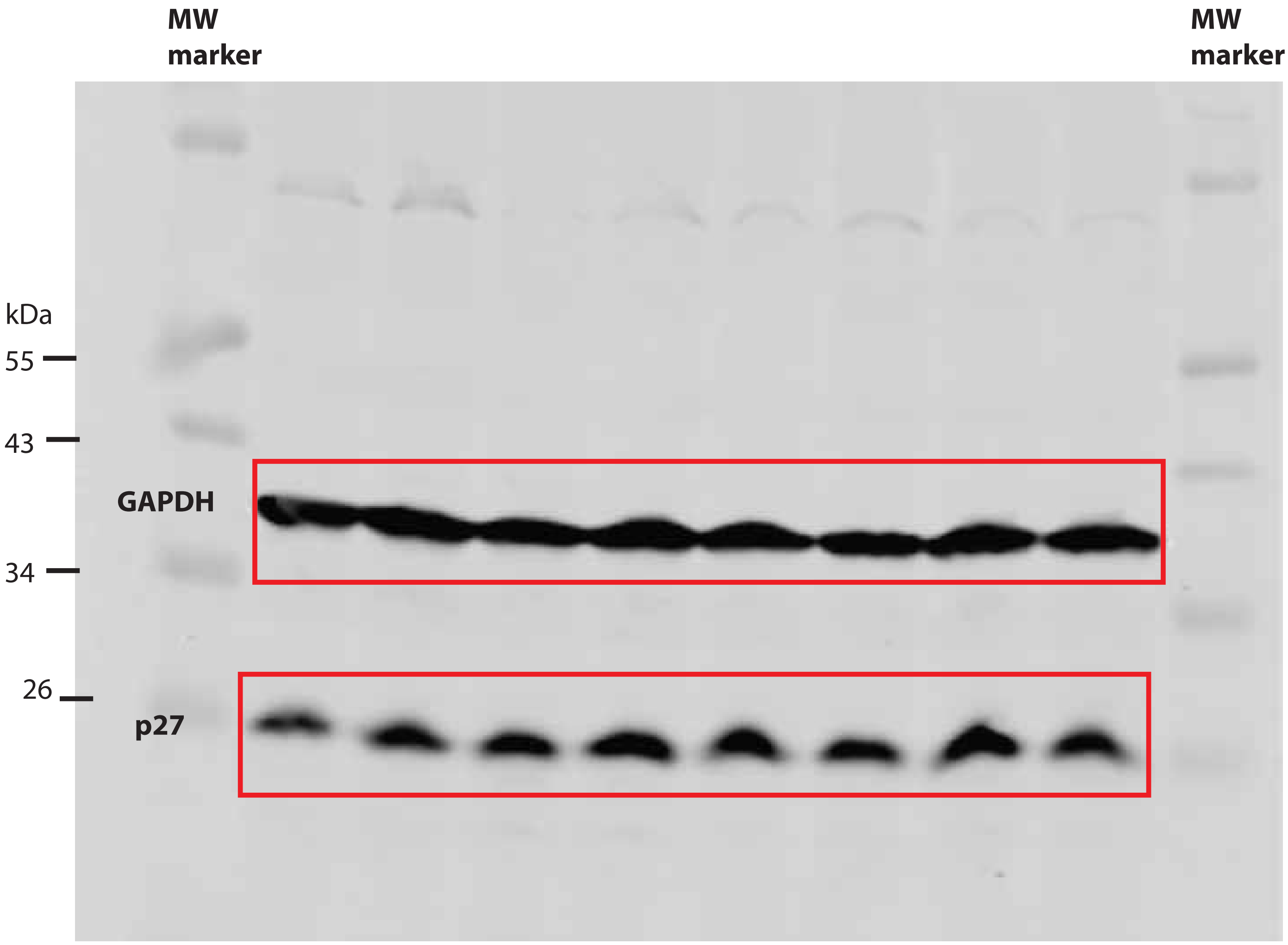

Western blot data for supplemental figure 6C
