# Supplementary material for: Siah2 control of T-regulatory cells limits anti-tumor immunity
Source: Nat Commun. 2020 Jan 7;11:99. doi: 10.1038/s41467-019-13826-7 (PMC6946684; doi:10.1038/s41467-019-13826-7)
Supplement: Supplementary file 2 — Reporting Summary [file 41467_2019_13826_MOESM2_ESM.pdf]

nature research

Corresponding author(s): Ze'ev Ronai

Last updated by author(s): Nov 5, 2019

Reporting Summary

Nature Research wishes to improve the reproducibility of the work that we publish. This form provides structure for consistency and transparency in reporting. For further information on Nature Research policies, seeAuthors & Referees and theEditorial Policy Checklist .

Statistics

For all statistical analyses, confirm that the following items are present in the figure legend, table legend, main text, or Methods section.

n/a	Confirmed	
☐	☒	The exact sample size (*n*) for each experimental group/condition, given as a discrete number and unit of measurement
☐	☒	A statement on whether measurements were taken from distinct samples or whether the same sample was measured repeatedly
☐	☒	The statistical test(s) used AND whether they are one- or two-sided<br>*Only common tests should be described solely by name; describe more complex techniques in the Methods section.*
☐	☒	A description of all covariates tested
☐	☒	A description of any assumptions or corrections, such as tests of normality and adjustment for multiple comparisons
☐	☒	A full description of the statistical parameters including central tendency (e.g. means) or other basic estimates (e.g. regression coefficient) AND variation (e.g. standard deviation) or associated estimates of uncertainty (e.g. confidence intervals)
☐	☒	For null hypothesis testing, the test statistic (e.g. *F*, *t*, *r*) with confidence intervals, effect sizes, degrees of freedom and *P* value noted<br>*Give P values as exact values whenever suitable.*
☒	☐	For Bayesian analysis, information on the choice of priors and Markov chain Monte Carlo settings
☒	☐	For hierarchical and complex designs, identification of the appropriate level for tests and full reporting of outcomes
☒	☐	Estimates of effect sizes (e.g. Cohen's *d*, Pearson's *r*), indicating how they were calculated

Our web collection on statistics for biologists contains articles on many of the points above.

Software and code

Policy information about availability of computer code

Data collection	All raw data of RNAseq, NanoString, and single cell RNAseq were deposited in the GEO platform and the respective accession numbers are noted in the manuscript.
Data analysis	Data were analyzed using GraphPad Prism 6 and 7 (graphing, statistical analysis), FlowJo v10.1 and v9.9 (flow cytometry), nSolver Analysis Software (Nanostring data analysis).

For manuscripts utilizing custom algorithms or software that are central to the research but not yet described in published literature, software must be made available to editors/reviewers. We strongly encourage code deposition in a community repository (e.g. GitHub). See the Nature Research guidelines for submitting code & software for further information.

Data

Policy information about availability of data

All manuscripts must include a data availability statement. This statement should provide the following information, where applicable:

- Accession codes, unique identifiers, or web links for publicly available datasets
- A list of figures that have associated raw data
- A description of any restrictions on data availability

All the data are available within the article, supplementary information, source data, and supplementary data file or from the authors upon request

Field-specific reporting

Please select the one below that is the best fit for your research. If you are not sure, read the appropriate sections before making your selection.

☒ Life sciences ☐ Behavioural & social sciences ☐ Ecological, evolutionary & environmental sciences

Life sciences study design

All studies must disclose on these points even when the disclosure is negative.

Sample size	No statistical methods were used to predetermine sample size. For in vivo work, the number of animal used in each experiment was carefully estimated based on our preliminary experiments and previous experience. For in vitro experiments, a minimum of 3 samples were chosen as a sample size to ensure adequate power, unless stated otherwise.
Data exclusions	Samples that had undergone technical failure during processing were excluded from analyses.
Replication	Experiments were repeated at least 2 or 3 times independently (unless otherwise stated) and were reproducible.
Randomization	Samples were not randomized. Mice were chosen based on their genotype.
Blinding	The investigators were not blinded to group allocation during experiments and outcome assessment.

Reporting for specific materials, systems and methods

We require information from authors about some types of materials, experimental systems and methods used in many studies. Here, indicate whether each material, system or method listed is relevant to your study. If you are not sure if a list item applies to your research, read the appropriate section before selecting a response.

Materials & experimental systems

n/a	Involved in the study	
☐	☒	Antibodies
☐	☒	Eukaryotic cell lines
☒	☐	Palaeontology
☐	☒	Animals and other organisms
☒	☐	Human research participants
☒	☐	Clinical data

Methods

n/a	Involved in the study	
☒	☐	ChIP-seq
☐	☒	Flow cytometry
☒	☐	MRI-based neuroimaging

Antibodies

Antibodies used

Biolegend: CD45.2 (104), CD8a (53-6.7), CD4 (GK1.5), CD45.1 (A20), TNFa (MP6-XT22), IFNg (XMG1.2), CD11c (N418), CD11b (M1/70), MHC class I (AF6-88.5) , CD80 (16-10A1), FOXP3 (FJK-16s), CXCR3 (CXCR3-173), CCR8 (SA214G2), CCR4 (2G12), GR1 (RB6-8C5), CD206 (C068C2), Thy1.1 (OX-7), Thy1.2 (53-21), KI67 (16A8), TGFb (TW7-16B4), IL10 (JES516E3), GITR (DTA-1), TBET (4B10), and CD25 (3C7)
BD Biosciences: GRANZYME B (GB11), NOS2 (CXNFT)
Cell Signaling Technology: Cleaved caspase 3 (9661), GAPDH (5174), p27 (3688)
Abcam: Ki67 (ab15580)
Invitrogen: Foxp3 (FJK-16S)
Santa Cruz: p27 (SC-528)
Miltenyi Biotec Inc: IFNGR1 (130-104-934)
BD Biosciences : BrdUflow kit (BDB559619)
R&D: anti-CCL17 (110904), anti-CCL22(158132), rat IgG2 isotype control (54447)
Antibodies are also listed in the Methods section under their respective experimental method.

Validation

We chose these antibodies based on information provided in product data sheets, literature and following our own pilot studies.

Eukaryotic cell lines

Policy information about cell lines

Cell line source(s)	YUMM1.7, and YUMMER1.7 cell lines were kindly provided by Marcus Bosenberg (Yale University). The Jurkat cell line was purchased from ATCC. MC38 was purchased from Kerafast.
Authentication	Cell lines were obtained from verified sources, and authenticated in SBP Genomic Core and our laboratory.
Mycoplasma contamination	All the cell lines tested negative for mycoplasma contamination (MycoAlert-Lonza)
Commonly misidentified lines (See ICLAC register)	No commonly misidentified cell lines were used.

Animals and other organisms

Policy information about studies involving animals; ARRIVE guidelines recommended for reporting animal research

Laboratory animals	C57BL/6 WT and Siah2-/- mice were maintained and used in the vivarium of Sanford Burnham Prebys Medical Discovery Institute. All mice used were a C57BL/6 genetic background and 6–10-weeks-old male mice. All experimental animal procedures were approved by the Institutional Animal Care and Use Committee of Sanford Burnham Prebys Medical Discovery Institute and UNL.
Wild animals	No wild animals were used in this study.
Field-collected samples	This study did not involve samples collected from the field.
Ethics oversight	All experimental animal procedures were approved by the Institutional Animal Care and Use Committee of Sanford Burnham Prebys Medical Discovery Institute (Approval # 16-070, 17-0431).

Note that full information on the approval of the study protocol must also be provided in the manuscript.

Flow Cytometry

Plots

Confirm that:

- [ ] The axis labels state the marker and fluorochrome used (e.g. CD4-FITC).
- [x] The axis scales are clearly visible. Include numbers along axes only for bottom left plot of group (a 'group' is an analysis of identical markers).
- [x] All plots are contour plots with outliers or pseudocolor plots.
- [x] A numerical value for number of cells or percentage (with statistics) is provided.

Methodology

Sample preparation	Tumor-derived single-cell suspensions were washed twice with FACS staining buffer, fixed for 15 min with 1% formaldehyde in PBS, washed twice, and resuspended in FACS staining buffer. For intracellular cytokine staining, cells were resuspended in complete RPMI-1640 (containing 10 mM HEPES, 1% non-essential amino acids and L-glutamine, 1 mM sodium pyruvate, 10% heat-inactivated fetal bovine serum (FBS), and antibiotics) supplemented with 50 U/mL IL-2 (NCI), 1 mg/mL brefeldin A (BFA, Sigma), and incubated with phorbol myristate acetate (10 ng/ml) and ionomycin (0.5 ug/ml) for 16 h at 37°C. The cells were then fixed and permeabilized using a Cytofix/Cytoperm Kit (BD Biosciences) before staining. For nuclear staining cells were fixed and permeabilized using Foxp3 fixation/permeabilization solution (Thermo Fisher).
Instrument	All data were collected on an LSRFortessa (BD Biosciences)
Software	All data were collected on FACSDive Software (BD) and analyzed using FlowJo v10.1 and v9.9 Software (Tree Star).
Cell population abundance	N/A
Gating strategy	CD4+ T cell analysis: CD45.2+CD4+CD25-<br>CD8 T cell analysis: CD45.2+CD8+<br>DCs analysis : CD45.2+CD11C+<br>Macrophage analysis: CD45.2+CD11b+F4/80+<br>Treg analysis: CD45.2+CD4+CD25+Foxp3+; CD45.2+CD4+CD25+

- [x] Tick this box to confirm that a figure exemplifying the gating strategy is provided in the Supplementary Information.
